# Genetic Diversity of Native Provenance and Plantation Populations of *Pinus sylvestris* var. *mongolica* Based on SSR Markers and Morphological Traits

**DOI:** 10.1002/ece3.70567

**Published:** 2024-11-19

**Authors:** Mingyu Yin, Bo Wu, Yingjun Pang, Tana Wuyun

**Affiliations:** ^1^ Institute of Ecological Conservation and Restoration Chinese Academy of Forestry Beijing China; ^2^ Key Laboratory of Desert Ecosystem and Global Change National Forestry and Grassland Administration Beijing China; ^3^ Research Institute of Non‐Timber Forestry Chinese Academy of Forestry Zhengzhou Henan China; ^4^ State Key Laboratory of Tree Genetics and Breeding Beijing China

**Keywords:** genetic diversity, genetic structure, morphological variation, *Pinus sylvestris* var. *mongolica*, plantation degradation

## Abstract

*Pinus sylvestris* var. *mongolica* plays a crucial role in the ecological restoration and industrial raw material production of arid and semiarid regions in northern China. The widespread degradation of its near‐mature plantation (over 30 years) is a significant concern, having been a topic of interest in recent decades. In this study, the genetic diversity and population genetic structure were assessed using 12 simple sequence repeat (SSR) markers within 11 native provenance populations and eight plantation populations. Additionally, variations in eight morphological traits of their offspring were evaluated at three sites in northern China. The results revealed high genetic diversity and weak genetic differentiation among the native provenance populations. The mean number of alleles (*N*_a_), allelic richness (*A*_r_), expected heterozygosity (*H*_e_), and Shannon–Wiener diversity index (*I*) were 5.492, 4.679, 0.550, and 1.120, respectively, and the genetic differentiation coefficient (*F*_ST_) was 0.022. Significant population effects of tree height and height to live crown base (HCB), as well as interactions of population with site and block within site, were observed in tree height, HCB, stem diameter at breast height (DBH), stem volume (VOL), crown shape (CS), and disease grade (DG). The genetic diversity parameters based on SSR markers and breeding values of tree height, DBH, HCB, VOL, and stem form (SF) of plantation populations were found to be lower than those of native provenance populations. Significant positive correlations were identified between the mean effective number of alleles per locus (*N*_e_) and VOL as well as *H*_e_ and crown width (CW). Furthermore, the maximum temperature of the warmest month (BIO5) and the silt content (T_SILT) were identified as significant factors influencing genetic diversity parameters and morphological variation, respectively. The findings provide scientific support for the reduction of plantation degradation, the effective restoration, and the sustainable management of forests for this species.

## Graphical abstract

This study revealed that the genetic diversity parameters and breeding values of phenotypic traits in plantation provenance populations were lower than those observed in native provenance populations of *Pinus sylvestris* var. mongolica. The number of effective alleles and expected heterozygosity were significantly positively correlated with stem volume and crown width. This suggests that the reduction in genetic diversity may have caused the degradation of plantation forest growth and stability. The maximum temperature of the warmest month (BIO5) and soil silt content (T_SILT) represent pivotal environmental variables that exert a considerable influence on genetic diversity parameters and breeding values, respectively. These findings provide scientific support for the reduction in plantation degradation, the effective restoration, and the sustainable management of forests for this species.

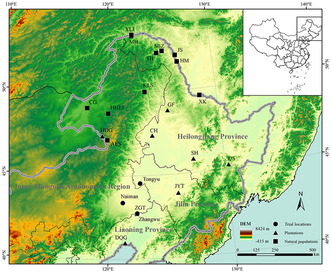

## 1 | Introduction

The degradation of plantations is frequently observed in ecologically sensitive regions. This is characterized by sluggish growth rates, diminished biomass, reduced canopy cover (Zhang et al. 2023), compromised stand stability (Wang and Shangguan 2022), and shorter life spans (Zhu et al. 2003), etc. These pose a significant risk to the ecological functionality and economic viability of the plantations. The causes of plantation degradation are thus of focal concern. Genetic diversity is a pivotal factor influencing the health and stability of populations, which usually differs between natural forests and plantations. For example, *Tectona grandis* showed lower genetic diversity of the plantation population in Java Island than that of the native provenance populations derived from the natural teak forests in India, Myanmar, Thailand, and Laos (Prasetyo et al. 2020). The reduction of genetic diversity in plantations can be explained by the fact that most plantations were established using plant materials from a limited number of mother trees or unsuitable provenances. For instance, *Quercus robur* plantations in Russia were established using clone seedlings or seeds sourced from a few selected superior mother trees, which resulted in homogeneous genetic compositions and low genetic variability of plantations (Degen et al. 2021). Moreover, environmental conditions also influence the genetic diversity of plantations. A case study conducted in Sweden indicated that young seedlings of *Picea abies* planted in nonforested and clear‐cut areas were subjected to more extreme environmental conditions, including drought, frost, and nutrient deficiencies, which increased the risk of genetic loss (Hu and Li 2002; Eriksson, Ekberg, and Clapham 2013).

In general, genetic diversity is a fundamental factor in the stability and functionality of forest ecosystems (Booy et al. 2000). It influences the evolutionary adaptability and resistance of tree populations to dynamic environmental conditions and biological disturbances (Rajora and Pluhar 2002). A rich genetic diversity maintains the health and stability of populations, whereas the loss of genetic variation can lead to the expression of deleterious recessive alleles due to increased homozygosity and inbreeding depression (Charlesworth and Willis 2009). A lower genetic diversity also influences the fitness and viability of populations to environmental shifts in the long term, and may consequently put such populations at an increased risk of local extinction (Manel and Holderegger 2013).

Molecular markers, such as simple sequence repeat (SSR) markers, have been extensively utilized in the genetic diversity evaluation of the aforementioned studies (Powell, Machray, and Provan 1996). Morphological characteristics are frequently determinants of the ecological and economic value, and thus are also commonly used to measure genetic diversity and to instruct breeders to select breeding material (Zhang et al. 2015). However, morphological traits are subject to modification by the influence of the environment and genotype‐by‐environment (G × E) interaction, which have been documented in several important coniferous tree species, including *P*. *taeda* (Li and McKeand 1989), *P*. *densiflora* (Kim et al. 2008), and *P*. *radiata* (Gapare et al. 2012). Significant G × E interactions might cause morphological performance of the same tree genotype to differ among different environments (Codesido and Fernández‐López 2009). The breeding value is the additive component of the genetic effect on the morphological traits. It is often calculated to eliminate the influence of these interactions and the environment (Cappa and Cantet 2008), and thus have been used to improve the efficiency and accuracy of genetic resource evaluation of trees (Li et al. 2017). Additionally, the identification of the environmental influences of native provenance on molecular markers‐based genetic diversity and morphological traits variation will facilitate a more comprehensive evaluation of germplasm resources and the effective introduction and silviculture of these resources.

The Mongolian pine ( *Pinus sylvestris* Linn. var. *mongolica* Litv.) is a geographical variant of the Scots pine ( *Pinus sylvestris* Linn.). It is indigenous to the northern Greater Khingan Mountains, the Hulunbuir Desert, the transition zone between the Greater and Lesser Khingan Mountains, and the banks of the Heilongjiang River in northeastern China, as well as parts of northeastern Mongolia and Russia (Zhu et al. 2003). The species is valued for its rapid growth, ecological benefits, and economic importance, exhibiting strong adaptability to drought, extreme cold (−40°C to −50°C), and poor soil fertility. Mongolian pine wood is renowned for its medium density, excellent stiffness and strength, and superior workability, and is therefore extensively utilized in furniture, flooring, house construction, and shipbuilding. Furthermore, the copious turpentine produced by this species is a vital resource for numerous industries, including medicine, healthcare, construction, environmental protection, and agriculture and forestry (Fernandes et al. 2017). Since its widespread introduction into arid and semiarid areas of northern China in the 1950s, Mongolian pine has played a pivotal role in windbreaks, soil and water conservation, ecological restoration, and timber supply, particularly in the “Three North” Protective Forest System Project of China. Approximately 800,000 ha of Mongolian pine plantations have been established. Nevertheless, the phenomena of degeneration, such as stunted growth and wilting leaves and branches, were frequently observed in the near‐mature plantations (over 30 years), which increased their vulnerability to pests and diseases.

To gain insight into the factors influencing the degradation processes of Mongolian pine plantations in comparison to their natural forests, extensive studies on tree physiology and plantation management have been conducted. These studies have focused on a range of topics, including stand structure and growth (Zhu et al. 2003), water and nutrient utilization (Han et al. 2020), and stand density regulation. Moreover, the majority of Mongolian pine plantations are established with seedlings derived from unselected germplasm, which has resulted in pronounced differentiation in stand growth and health. Previous studies have examined the genetic diversity of seven native provenance populations and two plantation populations of Mongolian pine using inter simple sequence repeat (ISSR) markers (Li et al. 2005), and analyzed the genetic structure of one native provenance population and three plantation populations using expressed sequence tag derived‐simple sequence repeat (EST‐SSR) markers from *P*. *tabuliformis* (Miao et al. 2018). Based on the results of the growth evaluation, three of the seven provenances were selected for applications in the Mao'ershan area of Heilongjiang Province, China (Xu, Sun, and Guo 2017). A comprehensive evaluation of genetic diversity for more natural and plantation populations, as well as germplasm selection for different environments, may provide a foundation for the mitigation of plantation degradation.

The present study investigated the growth performance of 31‐year‐old Mongolian pine in provenance trials conducted at three sites in northern China. The trials included 11 native provenances and eight secondary provenances (plantation sourced). Genetic diversity of these provenance populations was also assessed using SSR markers developed for the purpose. The objective of this study is to elucidate the SSR markers‐based genetic diversity and structure of native provenance populations and plantation populations, to characterize the morphological trait variations across three sites, and to examine the influence of climatic and soil factors on parameters of genetic diversity and morphological variations. The findings will contribute to a deeper understanding of the genetic diversity of Mongolian pine plantations, which will inform strategies for the conservation, improvement, and sustainable management of genetic resources for this species.

## 2 | Materials and Methods

### 2.1 | Overview of Provenance Trials

**FIGURE 1:**
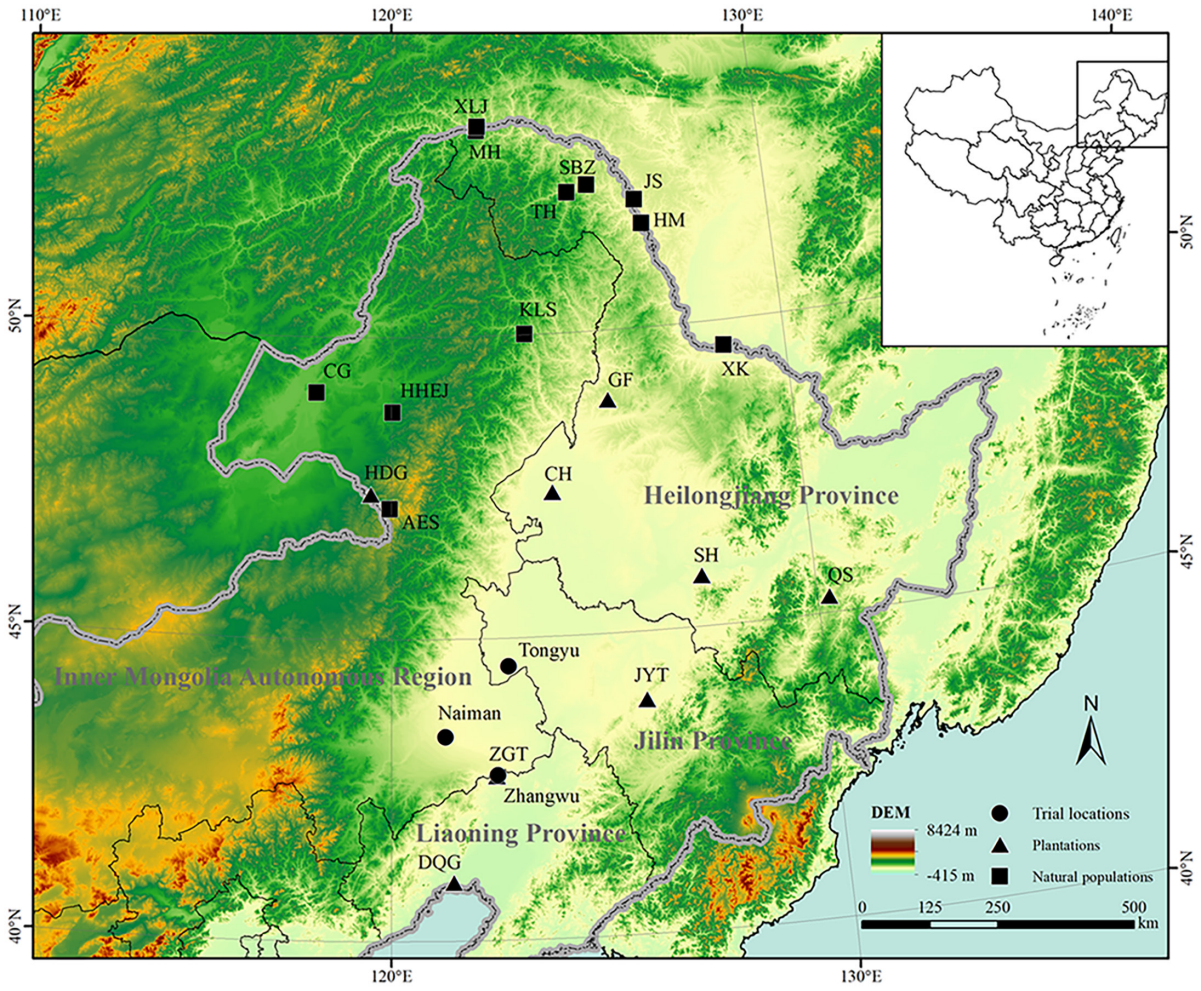
Locality of investigated plantations, natural populations, and provenance trials for *Pinus sylvestris* var. *mongolica*. See Table 1 for population abbreviations.

The provenance trials of Mongolian pine ( *Pinus sylvestris* var. *mongolica*) were conducted at three sites on the edge of the Horqin sandy plain (Figure 1): Zhangwu, Liaoning (ZL; N42°43′, E122°22′, 226.5 m a.s.l.); Naiman, Inner Mongolia (NIM; N43°22′, E122°14′, 241.0 m); and Tongyu, Jilin (TJ; N43°32′, E122°41′, 140.0 m) using its 2‐ or 3‐year‐old seedlings in 1992–1993. The mean annual precipitation (MAP) and mean annual air temperature (MAAT) were 514.0 mm and 6.9°C at ZL site, 343.3 mm and 6.1°C at NIM site, and 422.0 mm and 4.6°C at TJ site, respectively. The seedlings were raised with the seeds collected from 11 natural populations and eight plantations in the main distribution regions of China (Figure 1). At Site ZL, 19 provenances were involved, and 14 provenances for Sites NIM and TJ, the information are presented in Table 1. A randomized complete block design was implemented with 3, 4, and 4 replicates and 25, 16, and 25 trees per plot at ZL, NIM, and TJ sites, respectively. The seedlings were planted with a spacing of 1.5 m by 3.0 m.

**TABLE 1:** General information for 19 investigated populations of *Pinus sylvestris* var. *mongolica*.

Origin	Population abbreviation	Locality	Latitude (N)	Longitude (E)	Altitude (m)	MAP (mm)	MAAT (°C)	Sample size for SSR analysis	Zhangwu site	Naiman site	Tongyu site
Natural population	AES	Aershan, Inner Mongolia	47°10′	119°58′	1026.5	445	−2.9	18	√		
	CG	Cuogang, Inner Mongolia	49°05′	118°07′	588.0	284	−0.2	30	√	√	√
	HHEJ	Honghua'erji, Inner Mongolia	48°46′	120°02′	738.7	371	−1.6	26	√	√	√
	HM	Huma, Heilongjiang	51°43′	126°39′	177.4	487	−0.7	30	√	√	√
	JS	Jinshan, Heilongjiang	52°07′	126°31′	350.0	486	−1.3	15	√		
	KLS	Kalunshan, Heilongjiang	50°01′	123°25′	165.8	501	−1.2	30	√	√	√
	MH	Mohe, Heilongjiang	53°24′	122°20′	340.0	443	−4.2	25	√		
	SBZ	Shibazhan, Heilongjiang	52°25′	125°16′	350.0	500	−2.1	30	√	√	√
	TH	Tahe, Heilongjiang	52°19′	124°43′	357.0	501	−2.4	30	√	√	√
	XK	Xunke, Heilongjiang	49°35′	128°27′	111.9	501	−2.4	29	√	√	√
	XLJ	Xilinji, Heilongjiang	53°28′	122°22′	290.0	424	−4.1	29	√	√	√
Plantation	CH	Cuohai, Heilongjiang	47°23′	123°55′	150.0	423	3.7	30	√	√	√
	DQG	Daqinggou, Inner Mongolia	40°58′	121°22′	285.0	567	8.9	19	√		
	GF	Gaofeng, Heilongjiang	48°51′	125°25′	305.0	494	0.5	30	√	√	√
	HDG	Handagai, Inner Mongolia	47°24′	119°30′	642.0	373	−1.7	17	√		
	JYT	Jingyuetan, Jilin	43°52′	125°51′	300.0	632	4.2	30	√	√	√
	QS	Qingshan, Heilongjiang	45°16′	130°16′	273.6	565	3.5	30	√	√	√
	SH	Shihe, Heilongjiang	45°50′	127°21′	134.1	546	3.8	30	√	√	√
	ZGT	Zhanggutai, Liaoning	42°43′	122°22′	226.5	514	6.9	30	√	√	√

Abbreviations: MAAT, mean annual air temperature; MAP, mean annual precipitation.

### 2.2 | SSR Analysis

#### 2.2.1 | Leaf Sample Collection and DNA Extraction

For studies on population genetic diversity, a total of 508 individuals from 19 populations of Mongolian pine were sampled at ZL site in 2021. The sampling was conducted with the approval of and under the direction of the Research Institute of Sand Control and Utilization and thus did not require a permit from any other department prior to its commencement. For each tree, fresh and young leaves were collected and dried rapidly using silica gel, and deposited in the herbarium of the Institute of Ecological Conservation and Restoration, Chinese Academy of Forestry, China. Total genomic DNA was extracted from the leaves using a modified cetyltrimethylammonium bromide (CTAB) method (Zeng et al. 2002). The concentration and quality of the DNA samples were determined using a NanoDrop 2000 spectrophotometer (Thermo Fisher Scientific Inc., Waltham, MA, USA) and 1.0% agarose gel electrophoresis.

#### 2.2.2 | Polymorphic SSR Markers Development and SSR Genotyping

To develop new simple sequence repeat (SSR) markers for *P*. *sylvestris* var. *mongolica*, total DNA from a single individual from native provenance population Honghua'erji was fragmented into 350 bp segments using ultrasonication. A paired‐end library was constructed using the NEBNext Ultra DNA library prep kit (New England BioLabs, E7370S), followed by sequencing on the Illumina HiSeq X Ten platform with PE150 mode. A total of 10 gigabases (Gb) of data were obtained. The NGS QC Toolkit version 2.3.2 was used for quality control. Contigs were assembled from the high‐quality paired‐end reads using the SPAdes 3.6.1 program (Bankevich et al. 2012) with a k‐mer size of 95. In total, 6429 SSR markers were identified using the Genome‐Wide Microsatellite Analyzing Tool Package (GMATA) software (Wang and Wang 2016), utilizing search parameters set to a minimum of 5, 4, 3, 3, and 3 repeats for di‐, tri‐, tetra‐, penta‐, and hexanucleotide sequences, respectively, and 169 SSRs were then randomly selected for polymorphism testing. Meanwhile, four SSRs and 10 EST‐SSRs for *P*. *sylvestris* (Belletti et al. 2012; Sebastiani et al. 2012), and ten EST‐SSRs for *P*. *tabuliformis* (Fang et al. 2014), were detected for their transportable in Mongolian pine.

Polymerase chain reaction (PCR) was performed using the fluorescence‐labeled dUTP method. The forward primers of each maker were labeled at the 5′ end with an M13 sequence (5′‐CACGACGTTGTAAAACGAC‐3′). The M13 primer was also labeled at 3′ end with fluorescent dye FAM, VIC, NED, or ROX. The 10 μL PCR reaction system contained 5 ng DNA, 0.5 μM forward M13‐labeled primers, 0.5 μM reverse primers, 0.5 μM fluorescent M13 primers, 150 μM dNTPs, 2.0 μM MgCl_2_, 1 × PCR buffer (Tiangen Biotech Ltd., Beijing, China), and 0.25 U *Taq* DNA polymerase (Tiangen Biotech Ltd., Beijing, China). The PCRs were carried out on a Veriti thermal cycler (Applied Biosystems, Foster City, California, USA) under the following program: initial denaturation at 94°C for 4 min; followed by 31 cycles at 94°C for 30 s, annealing temperature for 30 s, and 72°C for 30 s (31 cycles); and a final 72°C for 10 min. The optimized annealing temperature was determined as 55°C through a PCR trial with a temperature gradient. The PCR products were analyzed by ABI 3730XL DNA Analyzer (Applied Biosystems Inc. USA) with GeneScan 500 LIZ internal size standard (Applied Biosystems Inc., USA). Genotyping of the SSR markers was performed using GeneMarker version 2.2 (Liu et al. 2011).

### 2.3 | Morphological Measurement

The growth performance was measured for all trees in each plot of the three provenance trials in July 2021. The measurements in Zhangwu, Naiman, and Tongyu were conducted with the approval of and under the direction of the Liaoning Research Institute of Sand Control and Utilization, Naiman Country Xinglongzhao State Mechanical Forest, and Tongyu County Second Mechanical Forestry, respectively. Consequently, no further permits were required from other departments prior to the commencement of the measurements. The diameter at breast height (DBH) was measured with a diameter tape (0.1 cm), tree height and height to live crown base (HCB) were measured with a Vertex IV Altimeter (Haglöf Sweden AB, Västernorrland, Sverige; 0.1 m), and crown width (CW) was measured with a sliding staff (0.1 m). Stem form (SF), branchiness (BRA), crown shape (CS), and disease grade (DG) were assessed and standardized following the methodologies outlined by Yin et al. (2019). The stem volume (VOL) was calculated using the following formula (Zhang et al. 2004):

(1) $$V=0.000115923\times {\mathrm{DBH}}^{1.917025106}H^{0.625080160}$$

### 2.4 | Data Analysis

Based on the SSR data set, the mean number of alleles (*N*_a_), the mean effective number of alleles (*N*_e_), observed heterozygosity (*H*_o_), expected heterozygosity (*H*_e_), Shannon–Wiener diversity index (*I*), polymorphic information content (*PIC*), and fixation index (*F*_IS_) were estimated for all populations. Deviations from Hardy–Weinberg equilibrium (HWE) were tested for each locus; Wright's *F*‐statistics, gene flow (*N*_m_), and Nei's genetic distances between the populations were assessed; and analysis of molecular variation (AMOVA) was performed for the native provenance populations, employing an infinite allele model with 999 permutations. These were all conducted using GenAlEx version 6.51 software (Peakall and Smouse 2012). The significance of *F*_IS_ was tested using a randomization procedure and a Bonferroni correction for multiple comparisons, and the 95% confidence intervals for the overall *F*_ST_ were also estimated in FSTAT version 2.9.3 software (Goudet 1995). Allelic richness (*A*_R_) for all populations and private allelic richness (*A*_P_) for the native provenance populations were calculated for standardized samples of 30 gene copies as the smallest sample size was 15 using HP‐RARE 1.0 software (Kalinowski 2005). The probability of occurrence of any recent bottleneck events was estimated in each native provenance population using BOTTLENECK version 1.2.0.2 software (Piry, Luikart, and Cornuet 1999), and the sign test, standardized differences test, and Wilcoxon's sign‐rank test were carried out under two‐phase model (TPM) with 1000 simulation iterations. The population genetic structure was examined through the Bayesian clustering method in STRUCTURE 2.3.3 software (Porras‐Hurtado et al. 2013). STRUCTURE was run 10 times for each defined K (2–19), with the length of initial burn‐in and the iteration of the Markov Chain Monte Carlo both 100,000. The STRUCTURE results were uploaded to STRUCTURE HARVESTER to visualize and determine the optimal delta K. The STRUCTURE results of multiple runs were averaged using CLUMPP software (Jakobsson and Rosenberg 2007), then Origin 2018 software (Moberly, Bernards, and Waynant 2018) for plotting.

Since there existed 14 common provenances at three sites (see Table 1), 14 provenances were involved in the analyses for morphological traits. Variance analysis was performed to evaluate variance components of traits with linear mixed model, considering site and block within site as fixed effects, and population, population–site, and population–block within site as random effects. Subsequently, the breeding values of morphological traits were estimated for these populations using the best linear unbiased prediction (BLUP) methodology by Genstat software, 18th edition (Yin et al. 2019).

The differences in genetic diversity parameters based on SSR markers and breeding values of morphological traits between native provenance and plantation populations were assessed using Cohen's *d* effect size with a set of widely used benchmark values (0.2 small, 0.5 medium, and 0.8 large) (Durlak 2009). The correlation between the genetic diversity parameters and the breeding values of morphological traits was analyzed using Pearson's coefficient. Mantel test was used to explore the relationship between distance matrices of the SSR markers and breeding values of morphological traits with GenAlEx version 6.51 software.

The influence of local environmental factors in native provenances on the variations of genetic diversity parameters and the breeding values of morphological traits were examined through redundancy analysis (RDA) using Canoco 5 software (Lepš and Šmilauer 2014). The data of 20 climatic factors were derived from the Worldclim‐Global Climate Database version 1.4 for the period 1950–2000 (http://www.worldclim.org/) (Table S1), and the data of topsoil (0–30 cm) factors were from the Harmonized World Soil Database version 1.2 (http://www.fao.org/).

## 3 | Results and Discussion

### 3.1 | Genetic Diversity Based on SSR Markers

#### 3.1.1 | SSR Polymorphism

In this study, 11 polymorphic SSR loci were developed for *Pinus sylvestris* var. *mongolica*, and one screened out from those of its relatives (Table 2). A total of 97 alleles were obtained in 508 individuals from 19 populations across the 12 polymorphic SSR loci, and their frequencies were presented in Table S2. The mean number of alleles (*N*_a_) ranged from three at loci Ps10, Ps36, and Ps82 to 21 at Ps8 with means of 8.083 (Table 3). The mean effective number of alleles (*N*_e_), expected heterozygosity (*H*_e_), Shannon–Wiener diversity index (*I*), and polymorphic information content (*PIC*) ranged from 1.385, 0.278, 0.468, and 0.241 at Locus Ps82 to 9.740, 0.897, 2.427, and 0.889 at Ps163 with means of 3.328, 0.556, 1.189, and 0.512, respectively. The observed heterozygosity (*H*_o_) was lower than the expected heterozygosity (*H*_e_), and their fixation index (*F*_IS_) was above 0 at Ps8, Ps63, and Ps70, indicating their highly significant deficiency in heterozygotes. A highly significant excess homozygosity was found at Ps61.

**TABLE 2:** Description of 12 SSR markers for *Pinus sylvestris* var. *mongolica* in this study.

Locus name	Primer sequence 5′‐3′	Repeated motif	Allele expected size (bp)	Annealing temperature (*T*_a_, °C)	GenBank accession numbers
Ps8	F:TCAGAAATATGCCAGGGCTAC	(AT)_7_	161–207	50	PP234675
	R:TGAAAATGGTGCACAAGAGG				
Ps10	F:TGGAAACTCTCTCTCTCTCATAAGG	(CT)_7_	172–176	55	PP234676
	R:GCACACAGCCAGGGAAAA				
Ps23	F:GGATCACTTGTGAAGGAGGAA	(TG)_8_	164–180	55	PP234677
	R:CATGTGGCACCCATGTAACT				
Ps36	F:CACCTTGTATCGGTTGGTAATTAAG	(TAT)_7_	166–175	55	PP234678
	R:CCAGTTGAGCTAACCCTTGG				
Ps61	F:CCCCTCTGAGTGTGATCTTTCT	(CA)_7_	179–195	55	PP234679
	R:TAACCCAACCCCAAAATTCA				
Ps63	F:GTGCTTCTACAATTTTGTGAGTGTC	(TG)_7_	180–186	55	PP234680
	R:GGGCTCTCATGGACCAATAC				
Ps70	F:TGCGGAAGCAAAGATAGAAA	(CA)_8_	172–188	55	PP234681
	R:GTCAGTTGGGCTTCCAAAGA				
Ps82	F:CCCATGTTTGTCAAGGTTCA	(AG)_7_	186–190	55	PP234682
	R:AGCCACACCAAAACATTTCC				
Ps89	F:ACAAAAGCCTCCGGAAAATC	(AT)_7_	187–197	55	PP234683
	R:GCTATAGGTTCATTACATTCCCAAC				
Ps163	F:CCCAAACACCGAGATAACCA	(GA)_21_	201–237	50	PP234684
	R:CCCCTCTTCTTACATCCCATT				
Ps164	F:GCGTCCAGGGACAAATCATA	(CA)_22_	138–176	55	PP234685
	R:TGCAATCACATATCGCACCT				
lw_isotig 07383^a^	F:CAAACAAAAAACAGTCTGCA	(CAT)_8_	184–196	55	KF501190
	R:ATCGTCATCATCATCGTCAC				

^a^ Locus lw_isotig 07383 is developed from *P*. *tabuliformis* Fang et al. (2014).

**TABLE 3:** Allelic polymorphisms for 12 microsatellite markers in *Pinus sylvestris* var. *mongolica*.

Locus	*N*_a_	*N*_e_	*H*_o_	*H*_e_	*I*	*PIC*	*F*_IS_
Ps8	21	3.987	0.593	0.749	2.030	0.736	0.208***
Ps10	3	1.584	0.347	0.369	0.583	0.306	0.058
Ps23	6	2.771	0.604	0.639	1.202	0.572	0.056
Ps36	3	1.839	0.447	0.456	0.689	0.362	0.021
Ps61	6	1.614	0.439	0.380	0.718	0.34	−0.154***
Ps63	4	2.927	0.549	0.658	1.096	0.585	0.167***
Ps70	8	2.115	0.358	0.527	1.088	0.485	0.321***
Ps82	3	1.385	0.249	0.278	0.468	0.241	0.105
Ps89	6	1.652	0.411	0.395	0.730	0.350	−0.042
Ps163	16	9.740	0.845	0.897	2.427	0.889	0.058
Ps164	16	8.494	0.855	0.882	2.349	0.871	0.031
lw_isotig07383	5	1.783	0.435	0.439	0.886	0.411	0.009
Mean	8.083	3.324	0.511	0.556	1.189	0.512	0.070***

*Notes: F*_IS_, fixation index; *H*_e_, the expected heterozygosity; *H*_o_, the observed heterozygosity; *I*, Shannon–Wiener diversity index; *N*_a_, the number of alleles per locus; *N*_e_, the effective number of alleles per locus; *PIC*, polymorphism information content.

*** *p* < 0.001.

#### 3.1.2 | Genetic Diversity

Considering the 11 native provenance populations of Mongolian pine, their mean values of *N*_a_, *N*_e_, allelic richness (*A*_r_), *H*_o_, and *H*_e_ were 5.492, 3.114, 4.679, 0.517, and 0.550, respectively, which were notably lower than those reported for natural Scots pine populations in Italy (14.45, 9.37, 8.992, 0.670, and 0.863; Belletti et al. 2012). This could be explained by the fact that Mongolian pine is a geographical variety of Scots pine, and is distributed in the marginal zone of this species (Zhu et al. 2003). Nevertheless, the genetic diversity was high in Mongolian pine. This could be interpreted as the diverse landscapes of the Greater and Lesser Khingan Mountains and their neighboring areas, including the Songliao Plain, the riverbank along the Heilongjiang and Songhua Rivers, as well as the grasslands and the sandy terrain of the Hulunbuir Plateau in northeastern China, which may create complex microenvironment and promote rich genetic diversity in Mongolian pine. A high level of genetic diversity was also observed for *Larix gmelinii* (Zhang et al. 2013) and *Betula platyphylla*, and *B*. *ermanii* (Wang et al. 2019) in this region. Bottleneck analysis showed that there was no significant heterozygote deficiency across native provenance populations of Mongolian pine by sign tests, standardized differences tests, and Wilcoxon signed‐rank tests, and all populations showed normal L‐shaped distribution (Table S3). These suggested that no recent bottleneck events have occurred in these populations, which also contributes to the maintenance of genetic diversity.

The mean values of *N*_e_, *A*_r_, *H*_o_, *H*_e_, and *I* of the plantation populations were all lower than those in the native provenance populations (Table 4). Medium‐effect sizes were revealed for *H*_e_ and *I*, and small effect sizes for *N*_e_, *A*_r_, and *H*_o_. *H*_e_ exceeded *H*_o_ in the majority of the 11 native provenance populations (except JS) and eight plantation populations (except HDG). These reductions in genetic diversity of most plantation populations of Mongolian pine might be a cause of the observed plantation degradation in practice, and be attributed to the establishment of these forests using seed from a limited number of mother trees. For example, *P*. *thunbergii* seeds were often collected from a few dominant trees, whereas the mother trees with smaller diameters at breast height might have higher allelic diversity in their seed pools, as indicated by Iwaizumi et al. (2023). This selection approach could inadvertently lead to a narrowed genetic spectrum within plantations. In addition, these Mongolian pine plantations were often established in harsh environmental conditions such as sandy soils, low fertility, drought conditions, and frigid winter temperatures, which can reach −40°C to −50°C. These could lead to pronounced genotype attrition, especially of the rare alleles, as highlighted in previous studies (Aravanopoulos 2011; Eriksson, Ekberg, and Clapham 2013). The private allelic richness (*A*_p_) of native provenance populations was 0.046, and 13 of a total of 97 alleles were private alleles which appeared only in one native provenance population (Table S2). The *A*_p_ of native provenance populations XLJ, AES, and HHEJ were higher than other populations, and this might reflect their adaptability to low temperatures in winter, high altitude, and drought conditions, respectively. The obvious positive correlation between *A*_p_ and environmental factors such as T_GRAVEL was also confirmed by redundancy analysis (RDA) (shown in Section 3.3). Similar results on *Shorea parvifolia* indicated that the variation in the number of private alleles might be related to the long‐term adaptation of populations to geographic microenvironment (Widiyatno Indrioko et al. 2016). Hu and Li (2002) indicated that genetic diversity parameters are sensitive to environmental shifts, especially of rare and private alleles, which are closely related to the health and stability of populations and are crucial for a tree species to adapt to environmental change.

**TABLE 4:** Genetic diversity parameters for 11 native provenance populations and eight plantation populations of *Pinus sylvestris* var. *mongolica*.

Origin	Population abbreviations	*N*_a_	*N*_e_	*A*_r_	*A*_p_	*H*_o_	*H*_e_	*I*	*F*_IS_
Native provenance population	AES	4.750	3.056	4.460	0.084	0.566	0.577	1.123	0.013
	CG	5.917	3.088	4.732	0.009	0.536	0.540	1.116	0.005
	HHEJ	5.417	3.039	4.569	0.072	0.439	0.547	1.100	0.182***
	HM	6.000	3.206	4.899	0.043	0.527	0.560	1.161	0.052*
	JS	4.833	2.958	4.692	0.058	0.566	0.559	1.111	−0.045
	KLS	5.667	3.190	4.794	0.041	0.517	0.557	1.151	0.040**
	XK	5.500	3.174	4.618	0.033	0.526	0.563	1.136	0.076**
	MH	5.500	3.159	4.686	0.011	0.487	0.530	1.093	0.058**
	SBZ	5.583	3.216	4.599	0.064	0.489	0.526	1.089	0.033*
	TH	5.417	3.120	4.581	0.002	0.503	0.531	1.092	0.045*
	XLJ	5.833	3.052	4.834	0.086	0.534	0.557	1.153	0.038*
Mean		5.492	3.114	4.679	0.046	0.517	0.550	1.120	0.046***
Plantation population	CH	6.250	3.233	4.928		0.527	0.551	1.158	
	DQG	5.000	3.121	4.555		0.535	0.562	1.118	
	GF	5.750	3.029	4.642		0.475	0.520	1.085	
	HDG	5.083	2.833	4.767		0.547	0.524	1.065	
	JYT	5.333	3.096	4.532		0.469	0.534	1.088	
	QS	5.667	3.247	4.792		0.490	0.542	1.127	
	SH	5.250	2.826	4.310		0.496	0.539	1.065	
	ZGT	5.667	3.076	4.641		0.533	0.533	1.101	
Mean		5.500	3.058	4.646		0.509	0.538	1.101	
Cohen's *d*		0.018	−0.448	−0.201		−0.247	−0.772	−0.667	

*Notes: A*r, allelic richness; *A*_p_, private allelic richness; *F*_IS_, fixation index; *H*_e_, the expected heterozygosity; *H*_o_, the observed heterozygosity; *I*, Shannon–Wiener diversity index; *N*_a_, the mean number of alleles per locus; *N*_e_, the mean effective number of alleles per locus.

*** *p* < 0.001.

** 0.001 ≤ *p* < 0.01.

* 0.01 ≤ *p* < 0.05, significant FIS value after Bonferroni correction (Bonferroni corrected *p* value, based on 2640 permutations, at *ɑ* = 0.05 was 0.00038).

#### 3.1.3 | Population Genetic Structure

**FIGURE 2:**
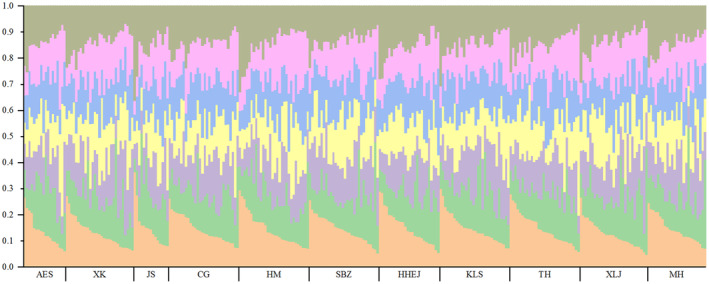
Genetic structure plot of native provenance populations of *Pinus sylvestris* var. *mongolica*. See Table 1 for population abbreviations.

The Chi‐square tests of Hardy–Weinberg equilibrium (HWE) for 11 native provenance populations showed that 26 of 132 tests showed significant deviations from HWE, and the number of loci deviating from HWE ranged from one in populations JS, KLS, and TH to four in population HHEJ (Table S4). The results of *F*‐statistics analysis showed that mean values of *F*_IS_, *F*_IT_, *F*_ST_, and gene flow (*N*_m_) across 12 loci were 0.046 (95% confidence interval [CI] –0.020 to 0.113), 0.068 (95% CI 0.002–0.133), 0.022 (95% CI 0.019–0.026), and 12.181 (95% CI 9.482–14.879), respectively. The significant *F*_ST_ indicated significant deficiency in heterozygotes within most populations, except AES, CG, and JS (Table 4). The *F*_ST_ was much lower than that in the previous studies on natural Scots pine populations in Italy (0.08, Scalfi et al. 2009; and 0.058, Belletti et al. 2012). These indicated frequent genetic exchange and low genetic differentiation among native provenance populations for Mongolian pine in northeastern China (detailed in Table S5). This was also confirmed by results of the analysis of molecular variance (AMOVA, Table 5), which showed that 99.55% of the genetic variation occurred within populations and only 0.45% among populations. STRUCTURE analysis showed that delta K was the largest when *K* = 7, and genetic structure plot (Figure 2) indicated genetic variation within populations was much greater than among them. Although the intricate terrain and climatic conditions in the Greater and Lesser Khingan Mountains regions result in a scattered and isolated distribution of Mongolian pine populations across mountains and sand areas, the characteristics of cross‐pollination and wind dispersal of pollen in Mongolian pine could be attributed to long‐distance gene flow among populations (see Table S5) and thus resulted in their homogeneous genetic composition. The long‐distance pollen dispersal usually plays an important role in mitigating fragmentation effect, which is also reported in Scots pine populations (Scalfi et al. 2009).

**TABLE 5:** Analysis of molecular variance of native provenance populations of *Pinus sylvestris* var. *mongolica*.

Source of variance	Degree of freedom	Sum of squares deviation	Mean squared deviation	Variance components	Total variation (%)	Genetic differentiation coefficient (*Phi*_PT_)
Among populations	10	83.283	8.328	0.033	0.45	0.004 (*p* = 0.061)
Within populations	281	2091.443	7.443	7.443	99.55	
Total	291	2174.726		7.476	100.00	

### 3.2 | Morphological Variation

Multisite variance analysis showed that the population effects were highly significant for tree height and height to crown base (HCB) at 0.001 level (Table 6). Interactions of population–site and population–block within site were significant for diameter at breast height (DBH), tree height, HCB, stem volume (VOL), crown shape (CS), and disease grade (DG) at the level of 0.05 or 0.001. Site and block effects were highly significant at 0.001 level for most of the traits except crown width (CW), CS, and branchiness (BRA). The variance component for morphological traits among populations accounted for 0.08% to 1.07%, and within populations for 76.22% to 99.21% of the total variation (Table 7). Similarly, variance component of interactions for population–site and population–block within site accounted for 0.13% to 1.71% and 0.68% to 23.57% of the total variation, respectively.

**TABLE 6:** Analysis of variance of morphological traits of *Pinus sylvestris* var. *mongolica* at three sites together.

Source of variance	df	DBH (cm)	Height (m)	HCB (m)	CW (m)	VOL (m^3^)	SF	CS	BRA	DG
Site	2	5756.73***	2528.73***	1260.94***	0.95	1.5505***	10.37***	4.26***	8.57***	1.07***
Block within site	8	47.79***	20.69***	11.99***	2.77***	0.0081***	0.38***	0.02	0.05	0.11***
Population	13	8.61	3.40***	2.07***	0.4	0.0019	0.07	0.05	0.08	0.04
Population–site	26	15.2***	2.52***	3.01***	0.43	0.0030***	0.10	0.07*	0.09	0.04*
Population–block Within site	98	12.26***	3.73***	2.18***	0.47	0.0030***	0.10	0.06*	0.07	0.04***
Error	1548	7.18	0.85	0.56	0.42	0.0013	0.08	0.05	0.07	0.03

Abbreviations: BRA, branchiness; CS, crown shape; CW, crown width; DBH, diameter at breast height; DG, disease degree; HCB, height to live crown base; SF, stem form; VOL, stem volume.

*** *p* < 0.001.

* 0.01 < *p* < 0.05, level of significance of effects.

**TABLE 7:** Estimation of variance components for population, population–site, and population–block within site of morphological traits for *Pinus sylvestris* var. *mongolica*.

Source of variance		DBH (cm)	Height (m)	HCB (m)	CW (m)	VOL (m^3^)	SF	CS	BRA	DG
Population	Variance component	0.0590	0.0024	0.0006	0.0005	0.0000	0.0003	0.0001	0.0005	0.0000
	Proportion to total variation (%)	0.77%	0.22%	0.08%	0.12%	1.07%	0.36%	0.17%	0.78%	0.14%
Population–site	Variance component	0.0260	NE	0.0126	NE	NE	NE	0.0001	0.0010	NE
	Proportion to total variation (%)	0.34%	NE	1.71%	NE	NE	NE	0.13%	1.40%	NE
Population–block within site	Variance component	0.4520	0.2622	0.1615	0.0029	0.0002	0.0018	0.0010	0.0012	0.0014
	Proportion to total variation (%)	5.86%	23.57%	21.98%	0.68%	10.34%	2.18%	2.23%	1.71%	5.08%
Error	Variance component	7.1750	0.8480	0.5600	0.4250	0.0013	0.0812	0.0455	0.0652	0.0265
	Proportion to total variation (%)	93.04%	76.22%	76.22%	99.21%	88.59%	97.46%	97.81%	96.11%	94.78%

*Notes:* See Table 6 for abbreviations of morphological traits; NE, Not estimated and assumed to be zero.

Previous studies have suggested that quality traits are generally under stronger genetic control than growth traits at the population level, such as stem straightness and branching characteristics of *P*. *radiate* showed high repeatability (Gapare et al. 2012). Similarly, for *B*. *alnoids*, repeatability was higher for stem form (SF), BRA, and HCB compared to growth traits like tree height, DBH, and individual VOL at the population level (Yin et al. 2019). Similar results in the present study showed that variance components of CS, BRA, SF, and DG (0.14%–0.78% with a mean of 0.36%) were higher than those of DBH, tree height, CW, and HCB (0.08%–0.77% with the mean of 0.29%) of Mongolian pine at the population level. Considering significant genotype‐by‐environment (G × E) interactions were revealed in tree height, DBH, HCB, VOL, CS, and DG, the genetic effect of these traits will be reduced. Such G × E interactions have been documented previously for traits such as tree height, DBH, SF, and VOL in *P*. *radiata* (Johnson and Buadon 1990; Falkenhagen 1996) and for tree height, DBH, and VOL in Masson pine ( *P*. *massoniana* ) (Yuan et al. 2021). These interactions indicated that genetic expression was also influenced by environmental factors, and inappropriate afforestation site selection might not maintain the good performance of excellent germplasm, which increased disease susceptibility, and potential degradation of Mongolian pine plantations.

The breeding values indicated the genetic effect of the morphological traits. Compared to plantation populations, breeding values for DBH, tree height, HCB, VOL, and SF were high in native provenance populations, with respective values of 0.0499, 0.0010, 0.0001, 0.0010, and 0.0045, respectively (Table 8). Conversely, CW, CS, BRA, and DG presented higher breeding values in plantation populations, with values of 0.0007, 0.0016, 0.0005, and 0.0000, respectively. Large, medium, and small negative effects were revealed for SF, DBH, and VOL of plantation populations compared to native provenance populations, respectively. These suggested that the current selection strategy of germplasm for plantation establishment may not achieve good growth and quality performance of these morphological traits, which cannot meet the management objectives of plantations in terms of ecological restoration and raw material production.

**TABLE 8:** Estimation of breeding values of morphological traits for eight native provenance populations and six plantation populations of *Pinus sylvestris* var. *mongolica*.

Origin	Population abbreviation	DBH	Height	HCB	CW	VOL	SF	CS	BRA	DG
Native provenance populations	KLS	0.0783	0.0063	−0.0016	0.0019	0.0007	0.0170	−0.0014	0.0245	0.0011
	SBZ	0.0816	−0.0119	−0.0067	−0.0004	0.0044	0.0207	−0.0027	0.0371	0.0016
	XK	−0.1301	−0.0034	0.0003	−0.0003	0.0001	−0.0079	0.0004	−0.0166	−0.0003
	XLJ	−0.1439	0.0131	0.0046	0.0023	−0.0042	−0.0196	−0.0019	−0.0140	0.0005
	HHEJ	−0.4092	0.0299	0.0077	0.0052	−0.0104	−0.0031	−0.0029	−0.0270	−0.0028
	TH	0.4233	−0.0093	−0.0010	−0.0205	0.0084	−0.0007	0.0026	−0.0186	0.0018
	HM	0.1757	−0.0122	−0.0029	0.0079	0.0042	0.0086	−0.0043	0.0105	−0.0025
	CG	0.3236	−0.0045	0.0003	−0.0004	0.0050	0.0212	0.0005	0.0014	0.0004
Mean		0.0499	0.0010	0.0001	−0.0005	0.0010	0.0045	−0.0012	−0.0003	0.0000
Plantation populations	CH	−0.0382	−0.0154	−0.0007	0.0111	0.0057	0.0019	−0.0022	−0.0076	0.0020
	GF	0.1914	−0.0100	0.0031	−0.0162	−0.0007	−0.0049	0.0092	−0.0228	0.0025
	JYT	−0.1688	−0.0047	−0.0026	−0.0007	0.0005	−0.0227	0.0011	0.0259	−0.0029
	SH	−0.2722	0.0187	−0.0034	0.0039	−0.0080	−0.0011	−0.0027	0.0157	−0.0005
	QS	−0.1347	−0.0143	−0.0012	0.0005	−0.0048	0.0003	0.0055	0.0296	0.0015
	ZGT	0.0232	0.0177	0.0042	0.0058	−0.0009	−0.0098	−0.0012	−0.0380	−0.0023
Mean		−0.0666	−0.0013	−0.0001	0.0007	−0.0014	−0.0060	0.0016	0.0005	0.0000
Cohen's *d*		−0.5215	−0.1518	−0.0525	0.1340	−0.4462	−0.8526	0.7476	0.0316	0.0354

*Notes:* See Table 1 for population abbreviations; and see Table 6 for abbreviations of morphological traits.

### 3.3 | Environmental Impact on Molecular and Morphological Variation

Genetic differentiation among Mongolian pine populations for the morphological traits (0.41%) was similar to that for the SSR markers (0.45%). The result of Mantel test showed a weak positive correlation (*r* = 0.245, *p* = 0.179) of distance matrices between breeding values of morphological traits and SSR markers. The study of Sharma et al. (2016) on *Hibiscus sabdariffa* indicated that genetic distances between majority populations based on morphological qualitative traits were greater than those based on SSR markers. These difference between morphological traits and SSR markers was also reported in the study of *Ziziphus jujuba* (Zhang et al. 2015). This could be attributed to that morphological variation and molecular divergence might be independent and respond to different evolutionary pressures (Wilson, Carlson, and White 1977).

**FIGURE 3:**
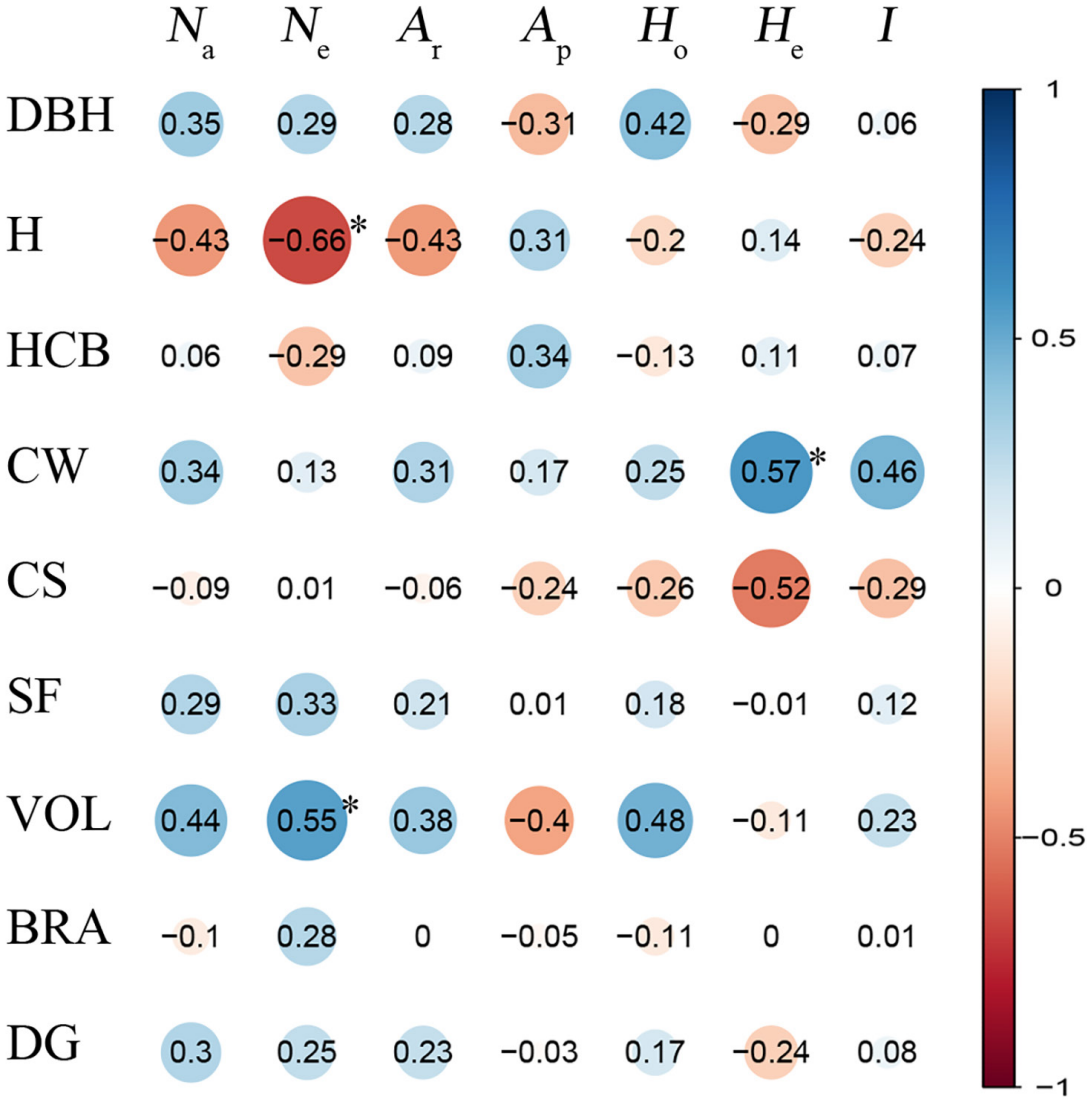
Correlation between SSR marker‐based genetic diversity parameters and breeding value of morphological traits of native provenance populations of *Pinus sylvestris* var. *mongolica*. See Tables 4 and 6 for abbreviations of SSR marker‐based genetic diversity parameters and morphological traits, respectively; *0.01 ≤ *p* < 0.05, level of significance.

The breeding values of morphological traits in Mongolian pine populations were closely related to genetic diversity parameters based on SSR loci (Figure 3). There were significant positive correlations between *N*_e_ and VOL, as well as *H*_e_ and CW (*p* < 0.05), and negative correlation between *N*_e_ and tree height. This indicated that genetic diversity played a key role in the growth and adaptability to environmental changes in populations. The benefits of greater genetic diversity are multifaceted, often characterized by enhanced population fitness and stable growth of individual trees (Reed and Frankham 2003). The previous study on *P*. *uncinata* revealed that high genetic diversity was instrumental in supporting the radial growth of individual trees under favorable climatic conditions (González‐Díaz et al. 2020). These insights underscore the importance of genetic diversity as a key factor in the growth and adaptability of Mongolian pine populations to environmental changes.

**FIGURE 4:**
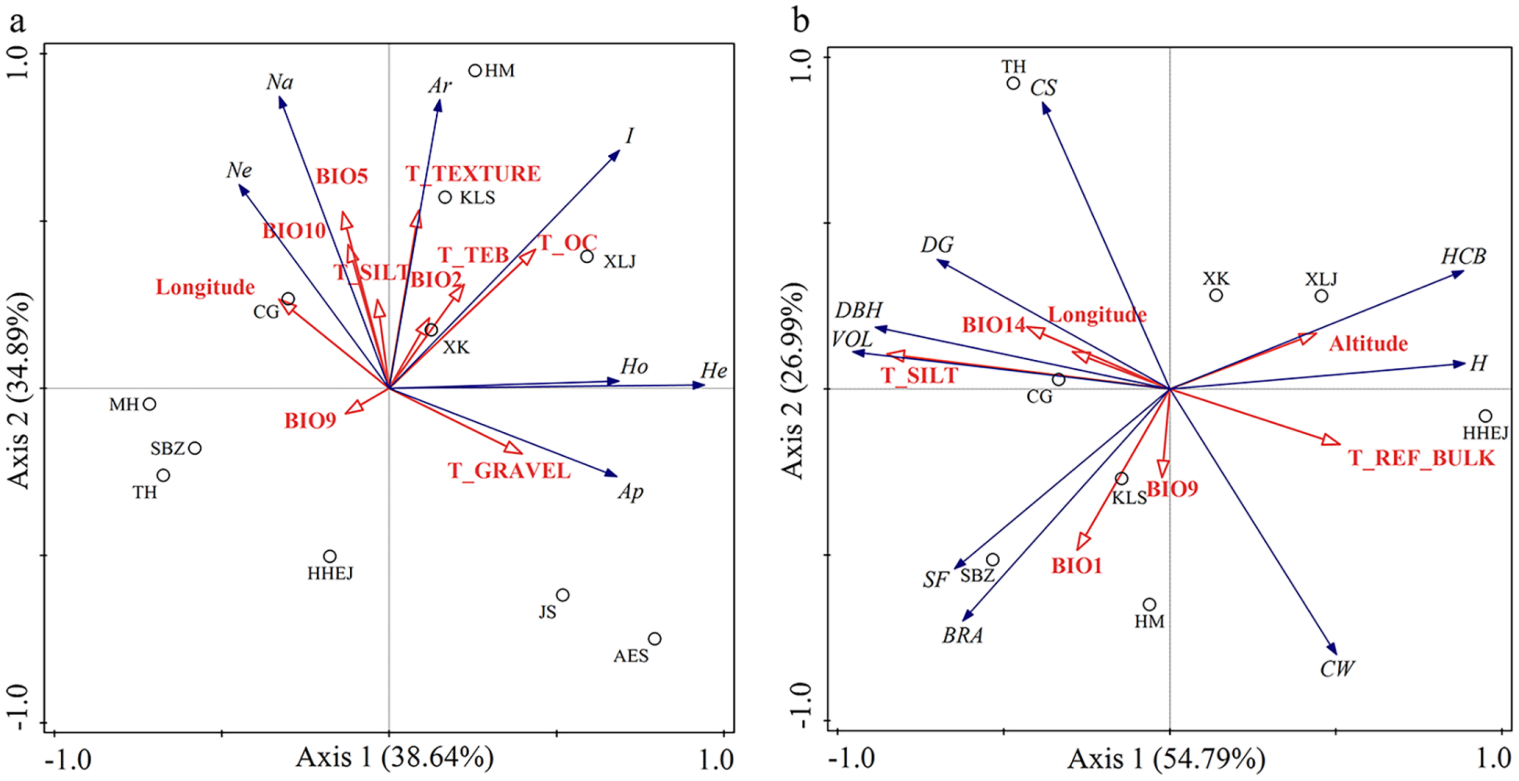
Redundancy analysis between climate and soil factors with genetic diversity parameters (a) and breeding value of morphological traits (b) of native provenance populations of *Pinus sylvestris* var. *mongolica*. See Table 1 for population abbreviations.

RDA results revealed that Axis 1 and Axis 2 explained a total of 73.53% of the overall variation of SSR markers‐based genetic diversity parameters (Figure 4a). BIO5 had the most significant influence on the variance of the genetic diversity parameters at the 0.05 level, with a contribution rate of 20.8%. The *N*_a_, *N*_e_, and *A*_r_ were positively correlated with BIO5, BIO10, T_SILT, longitude, and T_TEXTURE; the Shannon–Wiener diversity index (*I*) was positively correlated with T_OC, T_TEB, and BIO2; and the *A*_p_ was positively correlated with T_GRAVEL. This suggested that habitats with a higher proportion of silt, more organic carbon, greater nutrient levels, and higher summer temperatures, particularly those at higher longitudes, might support a rich allele pool and greater genetic diversity in native provenance populations of Mongolian pine. These effects of habitat environmental factors on genetic diversity are often reported for tree populations, such as summer seasonal precipitation was related to allele frequencies of 13 alpine plant species in the area of the European Alps (Manel et al. 2012). The *Q*. *petraea* populations from warmer and drier environments tended to lose diversity (Borovics and Mátyás 2013).

As for breeding value of morphological traits, RDA showed that Axis 1 and Axis 2 together accounted for 81.78% of the variation across native provenance populations (Figure 4b). T_SILT had the most significant influence on the variance of the breeding value at the 0.05 level, with a contribution rate of 44.1%. There was positive correlation of longitude, T_SILT, and BIO14 with traits such as VOL, DBH, DG, and CS. This suggested that native provenance populations from eastern regions of the natural distribution, characterized by higher winter rainfall and a higher percentage of silt in the soil, exhibited superior growth and health performance. There was positive correlation of tree height and HCB with altitude and T_REF_BULK, which demonstrated that native provenance population HHEJ, which originated from sandy areas at high altitudes, tended taller with higher HCB. Similarly, European populations of *P*. *sylvestris* from low‐altitude and high‐latitude locations were reported to have lower height growth (Wachowiak et al. 2017). The local adaptation of population to climate variability is one of the main processes shaping morphological variation (Vizcaíno‐Palomar et al. 2020), and the high correlations between trait variation and the geographical environment of origin were also observed in many studies. The tree height and radius of canopy showed highly significant differences among altitudinal ranges of *Terminalia catappa* in Indonesia (Marjenah and Putri 2017). The stem growth of five temperate broad‐leaved tree species was also affected by air temperature, precipitation, atmospheric water, and soil water (Köcher, Horna, and Leuschner 2012).

### 3.4 | Implications for Plantation Management of Mongolian Pine

The restoration of Mongolian pine in degraded and unproductive areas holds significant importance due to its impact on wood production and ecological benefits in northern China, as noted by Zhu et al. (2003). Investigating the discrepancies in SSR markers and morphological traits between native provenance populations and plantation populations offers insights into the underlying influence of genetic diversity on degradation of plantations. This understanding could inform the development of more effective strategies for forest restoration initiatives. Our findings reveal that genetic diversity based on SSR markers and morphological traits were independent, and SSR markers‐based genetic diversity parameters and breeding value of morphological traits (DBH, H, VOL, SF, and HCB) of plantation populations were obviously lower in comparison to native provenance populations. This inferred that the genetic diversity loss might be a cause for the degradation of Mongolian pine plantations. To counteract this, more genotypes should be collected in each provenance and delivered to reforestation programs. Additionally, since the significant environment effect and G × E interaction were observed in morphological traits, it was recommended that more genotypes and excellent provenances with good morphological performance in different environments should be included in afforestation to improve the genetic diversity and population stability of plantations. Native provenance population HM demonstrated the highest genetic diversity (*N*_a_, *A*_r_, and *I*), which should be given priority for conservation and utilization. Native provenance population TH and plantation population CH showed the best growth (DBH and VOL) and health performance (DG) across three sites, and could be applied preferentially so as to improve the health and stability of the plantations.

## 4 | Conclusions

The genetic differences between the plantation populations and the native provenance populations of *P*. *sylvestris* var. *mongolica* were evaluated based on the analysis of SSR markers and morphological traits. The results indicated that there was a high level of genetic diversity and a weak degree of genetic differentiation among native provenance populations based on SSR markers. Furthermore, it was observed that there were significant effects of site, population, and site–population interactions for the majority of morphological traits. Importantly, SSR markers‐based genetic diversity parameters and breeding values of most growth and quality traits in plantation populations were found to be lower than those observed in native provenance populations. Significant correlations were observed between genetic diversity parameters and the maximum temperature of the warmest month (BIO5) as well as between breeding values and soil silt content (T_SILT) for native provenance populations. These findings will facilitate the reduction in the degradation of plantations, the effective restoration of forests, and the management of germplasm for this species.

## Author Contributions

**Mingyu Yin:** data curation (equal), formal analysis (equal), investigation (equal), methodology (equal), software (equal), validation (equal), visualization (equal), writing – original draft (equal), writing – review and editing (equal). **Bo Wu:** conceptualization (equal), resources (equal), supervision (equal), validation (equal), visualization (equal), writing – original draft (equal), writing – review and editing (equal). **Yingjun Pang:** investigation (equal), project administration (equal), supervision (equal). **Tana Wuyun:** conceptualization (equal), investigation (equal), visualization (equal), writing – review and editing (equal).

## Conflicts of Interest

The authors declare no conflicts of interest.

## Supporting information


Table S1.
**Table S2**.
**Table S3**.
**Table S4**.
**Table S5**.

## Data Availability

The raw sequences of the SSR markers for Pinus sylvestris var. mongolica developed in this study can be obtained from NCBI Sequence Read Archive (GenBank accession numbers: PP234675–PP234685).

## References

[ece370567-bib-0001] Aravanopoulos, F. A. 2011. “Genetic Monitoring in Natural Perennial Plant Populations.” Botany 89, no. 2: 75–81. 10.1139/B10-087.

[ece370567-bib-0002] Bankevich, A. , S. Nurk , D. Antipov , et al. 2012. “SPAdes: A New Genome Assembly Algorithm and Its Applications to Single‐Cell Sequencing.” Journal of Computational Biology 19, no. 5: 455–477. 10.1089/cmb.2012.0021. 22506599 PMC3342519

[ece370567-bib-0003] Belletti, P. , D. Ferrazzini , A. Piotti , I. Monteleone , and F. Ducci . 2012. “Genetic Variation and Divergence in Scots Pine ( *Pinus sylvestris* L.) Within Its Natural Range in Italy.” European Journal of Forest Research 131, no. 4: 1127–1138. 10.1007/s10342-011-0584-3.

[ece370567-bib-0004] Booy, G. , R. J. J. Hendriks , M. J. M. Smulders , J. M. V. Groenendael , and B. Vosman . 2000. “Genetic Diversity and the Survival of Populations.” Plant Biology 2, no. 4: 379–395. 10.1055/s-2000-5958.

[ece370567-bib-0005] Borovics, A. , and C. Mátyás . 2013. “Decline of Genetic Diversity of Sessile Oak at the Retracting (Xeric) Limits.” Annals of Forest Science 70, no. 8: 835–844. 10.1007/s13595-013-0324-6.

[ece370567-bib-0006] Cappa, E. P. , and R. J. Cantet . 2008. “Direct and Competition Additive Effects in Tree Breeding: Bayesian Estimation From an Individual Tree Mixed Model.” Silvae Genetica 57, no. 1–6: 45–56. 10.1515/sg-2008-0008.

[ece370567-bib-0007] Charlesworth, D. , and J. H. Willis . 2009. “The Genetics of Inbreeding Depression.” Nature Reviews Genetics 10, no. 11: 783–796. 10.1038/nrg2664. 19834483

[ece370567-bib-0008] Codesido, V. , and J. Fernández‐López . 2009. “Implication of Genotype × Site Interaction on *Pinus radiata* Breeding in Galicia.” New Forest 37, no. 1: 17–34. 10.1007/s11056-008-9105-8.

[ece370567-bib-0009] Degen, B. , Y. Yanbaev , C. Blanc‐Jolivet , R. Ianbaev , S. Bakhtina , and M. Mader . 2021. “Genetic Comparison of Planted and Natural *Quercus robur* Stands in Russia.” Silvae Genetica 70, no. 1: 1–8. 10.2478/sg-2021-0001.

[ece370567-bib-0010] Durlak, J. A. 2009. “How to Select, Calculate, and Interpret Effect Sizes.” Journal of Pediatric Psychology 34, no. 9: 917–928. 10.1093/jpepsy/jsp004. 19223279

[ece370567-bib-0011] Eriksson, G. , I. Ekberg , and D. Clapham . 2013. Genetics Applied to Forestry an Introduction. 3rd ed, 1–206. Sverige AB: Elanders.

[ece370567-bib-0012] Falkenhagen, E. 1996. “A Comparison of the AMMI Method With Some Classical Statistical Methods in Provenance Research: The Case of the South African *Pinus radiata* Trails.” Forest Genetics 3, no. 2: 81–87.

[ece370567-bib-0013] Fang, P. , S. Niu , H. Yuan , et al. 2014. “Development and Characterization of 25 EST‐SSR Markers in *Pinus sylvestris* var. *Mongolica* (Pinaceae).” Applications in Plant Sciences 2, no. 1: 1300057. 10.3732/APPS.1300057. PMC4123385 25202597

[ece370567-bib-0014] Fernandes, C. , M. J. Gaspar , J. Pires , et al. 2017. “Physical, Chemical and Mechanical Properties of *Pinus sylvestris* Wood at Five Sites in Portugal.” International Forum for Environment, Sustainability & Technology 10, no. 4: 669–679. 10.3832/ifor2254-010.

[ece370567-bib-0015] Gapare, W. J. , M. Ivkovic , G. W. Dutkowski , D. J. Spencer , P. Buxton , and H. X. Wu . 2012. “Genetic Parameters and Provenance Variation of *Pinus radiata* D. Don. ‘Eldridge Collection’ in Australia 1: Growth and Form Traits.” Tree Genetics & Genomes 8, no. 2: 391–407. 10.1007/s11295-011-0449-4.

[ece370567-bib-0016] González‐Díaz, P. , A. Gazol , M. Valbuena‐Carabaña , et al. 2020. “Remaking a Stand: Links Between Genetic Diversity and Tree Growth in Expanding Mountain Pine Populations.” Forest Ecology and Management 472: 118244. 10.1016/j.foreco.2020.118244.

[ece370567-bib-0017] Goudet, J. 1995. “Fstat Version 1.2: A Computer Program to Calculate F Statistics.” Journal of Heredity 6: 485–486.

[ece370567-bib-0018] Han, H. , X. Zhang , H. Dang , et al. 2020. “Inter‐Annual Variation of Transpiration Intensity of *Pinus sylvestris* var. *Mongolica* Stand on the Southern Margin of Horqin Sandy Land and Its Relationship With Precipitation and Groundwater Level.” Scientia Silvae Sinica 56, no. 11: 31–40. 10.11707/j.1001-7488.20201104.

[ece370567-bib-0019] Hu, X. S. , and B. Li . 2002. “Linking Evolutionary Quantitative Genetics to the Conservation of Genetic Resources in Natural Forest Populations.” Silvae Genetica 51, no. 5–6: 177–184.

[ece370567-bib-0020] Iwaizumi, M. G. , A. A. Mukasyaf , I. Tamaki , et al. 2023. “Genetic Diversity and Structure of Seed Pools in an Old Planted *Pinus thunbergii* Population and Seed Collection Strategy for Gene Preservation.” Tree Genetics & Genomes 19, no. 9: 1–9. 10.1007/s11295-022-01584-5.

[ece370567-bib-0021] Jakobsson, A. , and N. A. Rosenberg . 2007. “CLUMPP: A Cluster Matching and Permutation Program for Dealing With Label Switching and Multimodality in Analysis of Population Structure.” Bioinformatics 23, no. 14: 1801–1806. 10.1093/bioinformatics/btm233. 17485429

[ece370567-bib-0022] Johnson, G. R. , and R. D. Buadon . 1990. “Family‐Site Interaction in *Pinus radiata* : Implications for Progeny Testing Strategy and Regionalised Breeding in New Zealand.” Silvae Genetica 39, no. 2: 55–62.

[ece370567-bib-0023] Kalinowski, S. T. 2005. “HP‐RARE 1.0: A Computer Program for Performing Rarefaction on Measures of Allelic Richness.” Molecular Ecology Notes 5, no. 1: 187–189. 10.1111/j.1471-8286.2004.00845.x.

[ece370567-bib-0024] Kim, I. S. , H. Y. Kwon , K. O. Ryu , and W. Y. Choi . 2008. “Provenance by Site Interaction of *Pinus densiflora* in Korea.” Silvae Genetica 57, no. 3: 131–139. 10.1515/sg-2008-0020.

[ece370567-bib-0025] Köcher, P. , V. Horna , and C. Leuschner . 2012. “Environmental Control of Daily Stem Growth Patterns in Five Temperate Broad‐Leaved Tree Species.” Tree Physiology 32, no. 8: 1021–1032. 10.1093/treephys/tps049. 22659458

[ece370567-bib-0026] Lepš, J. , and P. Šmilauer . 2014. Multivariate Analysis of Ecological Data Using Canoco 5. 2nd ed. Cambridge UK: Cambridge University Press. 10.2113/3.3.1057.

[ece370567-bib-0027] Li, H. , J. Jiang , G. Liu , X. Ma , J. Dong , and S. Lin . 2005. “Genetic Variation and Division of *Pinus sylvestris* Provenances by ISSR Markers.” Journal of Forestry Research 16, no. 3: 216–218. 10.1007/BF02856818.

[ece370567-bib-0028] Li, B. , and S. E. McKeand . 1989. “Stability of Loblolly Pine Families in the Southeastern US.” Silvae Genetica 38, no. 3–4: 96–101.

[ece370567-bib-0029] Li, Y. J. , M. Suontama , R. D. Burdon , and H. S. Dungey . 2017. “Genotype by Environment Interactions in Forest Tree Breeding: Review of Methodology and Perspectives on Research and Application.” Tree Genetics & Genomes 13, no. 60: 1–18. 10.1007/s11295-017-1144-x.

[ece370567-bib-0030] Liu, C. S. J. , D. Hulce , X. Li , and T. Snyder‐Leiby . 2011. “Genemarker® Genotyping Software: Tools to Increase the Statistical Power of DNA Fragment Analysis.” Journal of Biomolecular Techniques 22, no. Suppl: S35–S36.

[ece370567-bib-0031] Manel, S. , F. Gugerli , W. Thuiller , et al. 2012. “Broad‐Scale Adaptive Genetic Variation in Alpine Plants Is Driven by Temperature and Precipitation.” Molecular Ecology 21, no. 15: 3729–3738. 10.1111/j.1365-294X.2012.05656.x. 22680783 PMC4003392

[ece370567-bib-0032] Manel, S. , and R. Holderegger . 2013. “Ten Years of Landscape Genetics.” Trends in Ecology & Evolution 28, no. 10: 614–621. 10.1016/j.tree.2013.05.012. 23769416

[ece370567-bib-0033] Marjenah, M. , and N. P. Putri . 2017. “Morphological Characteristic and Physical Environment of *Terminalia catappa* in East Kalimantan, Indonesia.” Asian Journal of Forestry 1, no. 1: 33–39. 10.13057/asianjfor/r010105.

[ece370567-bib-0034] Miao, Y. , P. Fang , Z. Yang , et al. 2018. “Genetic Structure Analysis of *Pinus sylvestris* var. *Mongolica* Under Different Geographical Environments.” Journal of Beijing University 40, no. 10: 43–50. 10.13332/j.1000-1522.20170438.

[ece370567-bib-0035] Moberly, J. G. , M. T. Bernards , and K. V. Waynant . 2018. “Key Features and Updates for Origin 2018.” Journal of Cheminformatics 10, no. 5: 1–2. 10.1186/s13321-018-0259-x. 29427195 PMC5807254

[ece370567-bib-0036] Peakall, R. , and P. E. Smouse . 2012. “GenAlEx 6.5: Genetic Analysis in Excel. Population Genetic Software for Teaching and Research‐An Update.” Bioinformatics 28, no. 19: 2537–2539. 10.1093/bioinformatics/bts460. 22820204 PMC3463245

[ece370567-bib-0037] Piry, S. , D. Luikart , and J. M. Cornuet . 1999. “BOTTLENECK: A Computer Program for Detecting Recent Reductions in the Effective Population Size Using Allele Frequency Data.” Journal of Heredity 90, no. 4: 502–503. https://api.semanticscholar.org/CorpusID:18740868.

[ece370567-bib-0038] Porras‐Hurtado, L. , Y. Ruiz , C. Santos , C. Phillips , A. Carracedo , and M. V. Lareu . 2013. “An Overview of STRUCTURE: Applications, Parameter Settings, and Supporting Software.” Frontiers in Genetics 4: 98. 10.3389/fgene.2013.00098. 23755071 PMC3665925

[ece370567-bib-0039] Powell, W. , G. C. Machray , and J. Provan . 1996. “Polymorphisms Revealed by Simple Sequence Repeats.” Trends in Plant Science 1, no. 7: 215–222. 10.1016/1360-1385(96)86898-1.

[ece370567-bib-0040] Prasetyo, E. , S. Widiyatno Indrioko , M. Na'iem , et al. 2020. “Genetic Diversity and the Origin of Commercial Plantation of Indonesian Teak on Java Island.” Tree Genetics & Genomes 16, no. 34: 1–14. 10.1007/s11295-020-1427-5.

[ece370567-bib-0041] Rajora, O. P. , and S. A. Pluhar . 2002. “Genetic Diversity Impacts of Forest Fires, Forest Harvesting, and Alternative Reforestation Practices in Black Spruce ( *Picea mariana* ).” Theoretical and Applied Genetics 106, no. 7: 1203–1212. 10.1007/s00122-002-1169-9. 12748771

[ece370567-bib-0042] Reed, D. H. , and R. Frankham . 2003. “Correlation Between Fitness and Genetic Diversity.” Conservation Biology 17, no. 1: 230–237. 10.1046/j.1523-1739.2003.01236.x.

[ece370567-bib-0043] Scalfi, M. , A. Piotti , M. Rossi , and P. Piovani . 2009. “Genetic Variability of Italian Southern Scots Pine ( *Pinus sylvestris* L.) Populations: The Rear Edge of the Range.” European Journal of Forest Research 128: 377–386. 10.1007/s10342-009-0273-7.

[ece370567-bib-0044] Sebastiani, F. , F. Pinzauti , S. T. Kujala , S. C. González‐Martínez , and G. G. Vendramin . 2012. “Novel Polymorphic Nuclear Microsatellite Markers for *Pinus sylvestris* L.” Conservation Genetics Resources 4, no. 2: 231–234. 10.1007/s12686-011-9513-5.

[ece370567-bib-0045] Sharma, H. K. , M. Sarkar , S. B. Choudhary , et al. 2016. “Diversity Analysis Based on Agro‐Morphological Traits and Microsatellite Based Markers in Global Germplasm Collections of Roselle ( *Hibiscus sabdariffa* L.).” Industrial Crops and Products 89: 303–315. 10.1016/j.indcrop.2016.05.027.

[ece370567-bib-0046] Vizcaíno‐Palomar, N. , B. Fady , R. Alía , A. Raffin , S. Mutke , and M. B. Garzónet . 2020. “The Legacy of Climate Variability Over the Last Century on Populations' Phenotypic Variation in Tree Height.” Science of the Total Environment 749: 141454. 32814202 10.1016/j.scitotenv.2020.141454

[ece370567-bib-0047] Wachowiak, W. , A. Perry , K. Donnelly , and S. Cavers . 2017. “Early Phenology and Growth Trait Variation in Closely Related European Pine Species.” Ecology and Evolution 8, no. 1: 655–666. 10.1002/ece3.3690. 29321902 PMC5756864

[ece370567-bib-0048] Wang, Y. , and Z. Shangguan . 2022. “Formation Mechanisms and Remediation Techniques for Low‐Efficiency Artificial Shelter Forests on the Chinese Loess Plateau.” Journal of Arid Land 14, no. 8: 837–848. 10.1007/s40333-022-0069-x.

[ece370567-bib-0049] Wang, X. , and L. Wang . 2016. “GMATA: An Integrated Software Package for Genome‐Scale SSR Mining, Marker Development and Viewing.” Frontiers in Plant Science 7: 1350. 10.3389/fpls.2016.01350. 27679641 PMC5020087

[ece370567-bib-0050] Wang, H. , X. Yin , D. Yin , L. Li , and H. Xiao . 2019. “Population Genetic Structures of Two Ecologically Distinct Species *Betula platyphylla* and *B*. *ermanii* Inferred Based on Nuclear and Chloroplast DNA Markers.” Ecology and Evolution 9, no. 2: 11406–11419. 10.1002/ece3.5643. 31641482 PMC6802015

[ece370567-bib-0051] Widiyatno Indrioko, S. , M. Na'iem , K. Uchiyama , et al. 2016. “Effects of Different Silvicultural Systems on the Genetic Diversity of *Shorea Parvifolia* Populations in the Tropical Rainforest of Southeast Asia.” Tree Genetics & Genomes 12, no. 73: 1–12. 10.1007/s11295-016-1030-y.

[ece370567-bib-0052] Wilson, A. C. , S. S. Carlson , and T. J. White . 1977. “Biochemical Evolution.” Annual Review of Biochemistry 46: 573–639. 10.1146/annurev.bi.46.070177.003041 409339

[ece370567-bib-0053] Xu, J. , H. Sun , and B. Guo . 2017. “The Superior Population Selection of Mongolian Scotch Pine ( *Pinus sylvestris* var. *Mongolica*) Forests at Maoershan Area.” Journal of Nanjing Forestry University (Natural Sciences Edition) 41, no. 1: 61–68. 10.3969/j.issn.1000-2006.2017.01.010.

[ece370567-bib-0054] Yin, M. , J. Guo , C. Wang , Z. Zhao , and J. Zeng . 2019. “Genetic Parameter Estimates and Genotype × Environment Interactions of Growth and Quality Traits for *Betula alnoides* Buch.‐Ham. Ex D. Don in Four Provenance‐Family Trials in Southern China.” Forests 10, no. 11: 1036. 10.3390/f10111036.

[ece370567-bib-0055] Yuan, C. , Z. Zhang , G. Jin , et al. 2021. “Genetic Parameters and Genotype by Environment Interactions Influencing Growth and Productivity in Masson Pine in East and Central China.” Forest Ecology and Management 487: 118991. 10.1016/j.foreco.2021.118991.

[ece370567-bib-0056] Zeng, J. , Y. Zou , J. Bai , and H. Zheng . 2002. “Preparation of Total DNA From “Recalcitrant Plant Taxa”.” Acta Botanica Sinica 44, no. 6: 694–697. https://www.jipb.net/EN/Y2002/V44/I6/694.

[ece370567-bib-0057] Zhang, Z. , J. Gao , D. Kong , et al. 2015. “Assessing Genetic Diversity in *Ziziphus jujuba* ‘Jinsixiaozao’ Using Morphological and Microsatellite (SSR) Markers.” Biochemical Systematics and Ecology 61: 196–202. 10.1016/j.bse.2015.06.021.

[ece370567-bib-0058] Zhang, J. , Z. Li , W. Duan , et al. 2023. “Assessing Restoration and Degradation of Natural and Artificial Vegetation in the Arid Zone of Northwest China.” Frontiers in Ecology and Evolution 11: 1131210. 10.3389/fevo.2023.1131210.

[ece370567-bib-0059] Zhang, R. , H. Yu , G. Wang , W. Cao , F. Zhou , and X. Bai . 2004. “Preparation of a Binary Timber Volume Table for *Pinus sylvestris* var. *Mongolica* Plantations in Liaoning Province.” Journal of Liaoning Forestry Science & Technology & Technology 6: 22–24. (In Chinese).

[ece370567-bib-0060] Zhang, Z. , H. Zhang , J. Du , and L. Zhang . 2013. “RAPD and SSR Analysis of Genetic Diversity of Natural *Larix gmelinii* Populations.” Biotechnology & Biotechnological Equipment 27, no. 4: 3959–3965. 10.5504/BBEQ.2013.0059.

[ece370567-bib-0061] Zhu, J. , Z. Fan , D. Zeng , F. Jiang , and T. Matsuzaki . 2003. “Comparison of Stand Structure and Growth Between Artificial and Natural Forests of *Pinus sylvestiris* Var. *Mongolica* on Sandy Land.” Journal of Forestry Research 14, no. 2: 103–111. 10.1007/BF02856774.

