# Supplementary material for: Genetic Diversity of Native Provenance and Plantation Populations of Pinus sylvestris var. mongolica Based on SSR Markers and Morphological Traits
Source: Ecol Evol. 2024 Nov 19;14(11):e70567. doi: 10.1002/ece3.70567 (PMC11576128; doi:10.1002/ece3.70567)
Supplement: Supplementary file 1 — Table S1. Table S2. Table S3. Table S4. Table S5. [file ECE3-14-e70567-s001.docx]

**Appendix A. Supplementary material**

**TABLE S1** Information of 20 climate factors and 18 soil factors.

| Abbreviation | Climate factors | Unit |
| --- | --- | --- |
| Altitude | Altitude | m |
| BIO1 | Annual mean temperature | ℃ |
| BIO2 | Mean diurnal range | ℃ |
| BIO3 | Isothermality | % |
| BIO4 | Temperature seasonality | % |
| BIO5 | Max temperature of warmest month | ℃ |
| BIO6 | Min temperature of coldest month | ℃ |
| BIO7 | Temperature annual range | ℃ |
| BIO8 | Mean temperature of wettest quarter | ℃ |
| BIO9 | Mean temperature of driest quarter | ℃ |
| BIO10 | Mean temperature of warmest quarter | ℃ |
| BIO11 | Mean temperature of coldest quarter | ℃ |
| BIO12 | Annual precipitation | mm |
| BIO13 | Precipitation of wettest month | mm |
| BIO14 | Precipitation of driest month | mm |
| BIO15 | Precipitation seasonality | mm |
| BIO16 | Precipitation of wettest quarter | mm |
| BIO17 | Precipitation of driest quarter | mm |
| BIO18 | Precipitation of warmest quarter | mm |
| BIO19 | Precipitation of coldest quarter | mm |
| T_TEXTURE | Texture classification of top soil | none |
| AWC_CLASS | Available water content of top soil | mm |
| T_GRAVEL | Gravel volume percentage of top soil | %volume |
| T_SAND | Sand content of top soil | %weight |
| T_SILT | Silt content of top soil | %weight |
| T_CLAY | Clay content of top soil | %weight |
| T_USDA_TEX | Texture classification of top soil by United States Department Of Agriculture | none |
| T_REF_BULK | Soil reference bulk density of top soil | g/cm^3^ |
| T_OC | Organic carbon content of top soil | %weight |
| T_PH_H2O | Soil pH of top soil | –log(H^+^) |
| T_CEC_CLAY | Cation exchange capacity of clay layer soil of top soil | cmol·kg^-1^ |
| T_CEC_SOIL | Cation exchange capacity of the soil of top soil | cmol·kg^-1^ |
| T_BS | Basic saturation of top soil | % |
| T_TEB | Total exchangeable salt base of top soil | cmol·kg^-1^ |
| T_CACO3 | Carbonate content of top soil | %weight |
| T_CASO4 | Sulfate content of top soil | %weight |
| T_ESP | Exchangeable sodium salt of top soil | % |
| T_ECE | Electrical conductivity of top soil | dS·m^-1^ |

**TABLE S2** Allele frequencies in 19 populations of *Pinus sylvestris* var*. mongolica*.

| Locus | Allele | Native provenance populations | | | | | | | | | |  |  | Plantation populations | | | | | | | |
| --- | --- | --- | --- | --- | --- | --- | --- | --- | --- | --- | --- | --- | --- | --- | --- | --- | --- | --- | --- | --- | --- |
|  |  | AES | CG | HHEJ | HM | JS | KLS | MH | SBZ | TH | XK | XLJ |  | CH | DQG | GF | HDG | JYT | QS | SH | ZGT |
| Ps8 | a |  | 0.017 | 0.058 | 0.018 | 0.077 | 0.037 | 0.080 | 0.033 | 0.121 |  |  |  | 0.017 | 0.053 | 0.017 | 0.063 | 0.033 | 0.038 | 0.117 | 0.086 |
|  | b | 0.353 | 0.603 | 0.442 | 0.464 | 0.462 | 0.389 | 0.540 | 0.450 | 0.466 | 0.466 | 0.448 |  | 0.400 | 0.263 | 0.450 | 0.563 | 0.567 | 0.500 | 0.617 | 0.431 |
|  | c | 0.029 |  |  | 0.036 | 0.115 | 0.037 | 0.100 | 0.067 | 0.086 | 0.052 | 0.034 |  | 0.017 | 0.026 | 0.017 |  | 0.033 | 0.038 |  | 0.017 |
|  | d | 0.059 | 0.034 | 0.135 | 0.018 | 0.038 | 0.019 | 0.020 | 0.017 | 0.017 | 0.017 |  |  | 0.017 | 0.053 | 0.017 | 0.094 | 0.050 | 0.038 | 0.083 |  |
|  | e |  | 0.017 | 0.019 |  |  |  | 0.020 |  |  | 0.052 | 0.034 |  | 0.033 | 0.026 | 0.017 |  |  |  |  |  |
|  | f | 0.118 | 0.103 | 0.115 | 0.071 | 0.077 | 0.185 | 0.120 | 0.067 | 0.086 | 0.017 | 0.086 |  | 0.133 |  | 0.100 | 0.063 | 0.083 | 0.038 | 0.033 | 0.138 |
|  | g |  |  | 0.019 | 0.018 |  | 0.037 |  |  | 0.052 | 0.034 | 0.034 |  | 0.050 |  | 0.017 | 0.063 | 0.017 | 0.019 | 0.017 | 0.017 |
|  | h | 0.029 |  |  | 0.018 |  |  |  |  |  | 0.034 | 0.069 |  | 0.017 |  | 0.017 |  |  | 0.038 |  | 0.017 |
|  | i |  |  | **0.019** |  |  |  |  |  |  |  |  |  |  |  |  |  |  |  |  |  |
|  | j |  |  |  |  |  | **0.019** |  |  |  |  |  |  |  |  |  |  |  |  |  |  |
|  | k |  | 0.034 |  | 0.018 |  |  |  | 0.017 | 0.017 | 0.017 | 0.017 |  | 0.033 |  | 0.050 |  | 0.017 |  | 0.050 |  |
|  | l |  |  |  | 0.036 |  |  |  | 0.033 | 0.017 | 0.017 | 0.017 |  |  | 0.026 | 0.033 |  |  |  |  |  |
|  | m |  |  |  |  |  | 0.037 |  | 0.033 |  | 0.017 | 0.017 |  | 0.017 |  | 0.033 |  |  |  |  | 0.017 |
|  | n | 0.147 | 0.103 | 0.096 | 0.107 | 0.077 | 0.037 | 0.040 | 0.117 | 0.034 | 0.103 | 0.138 |  | 0.133 | 0.079 | 0.150 | 0.031 | 0.100 | 0.096 | 0.050 | 0.086 |
|  | o | 0.118 | 0.052 | 0.058 | 0.125 | 0.115 | 0.111 | 0.020 | 0.033 | 0.086 | 0.121 | 0.052 |  | 0.067 | 0.132 | 0.050 | 0.063 | 0.050 | 0.077 | 0.017 | 0.052 |
|  | p |  |  |  |  |  |  | 0.020 |  | 0.017 |  |  |  |  |  |  | 0.063 | 0.017 |  |  |  |
|  | q |  | 0.017 | 0.019 | 0.036 |  | 0.037 | 0.020 | 0.017 |  |  |  |  | 0.033 | 0.132 |  |  |  | 0.058 |  | 0.017 |
|  | r | 0.088 |  |  | 0.036 |  |  | 0.020 | 0.017 |  |  |  |  |  | 0.158 |  |  |  | 0.038 |  | 0.052 |
|  | s | **0.029** |  |  |  |  |  |  |  |  |  |  |  |  | 0.053 |  |  |  |  |  |  |
|  | t | 0.029 | 0.017 | 0.019 |  | 0.038 | 0.056 |  | 0.100 |  | 0.017 | 0.052 |  | 0.033 |  | 0.033 |  | 0.033 | 0.019 | 0.017 | 0.052 |
|  | u |  |  |  |  |  |  |  |  |  | **0.034** |  |  |  |  |  |  |  |  |  | 0.017 |
| Ps10 | a |  | 0.017 |  |  | 0.033 |  |  | 0.017 | 0.017 |  |  |  | 0.017 |  |  |  | 0.033 |  |  |  |
|  | b | 0.667 | 0.750 | 0.596 | 0.767 | 0.667 | 0.733 | 0.780 | 0.850 | 0.733 | 0.776 | 0.875 |  | 0.767 | 0.763 | 0.850 | 0.735 | 0.733 | 0.700 | 0.733 | 0.850 |
|  | c | 0.333 | 0.233 | 0.404 | 0.233 | 0.300 | 0.267 | 0.220 | 0.133 | 0.250 | 0.224 | 0.125 |  | 0.217 | 0.237 | 0.150 | 0.265 | 0.233 | 0.300 | 0.267 | 0.150 |
| Ps23 | a |  |  |  |  |  |  |  |  | **0.067** |  |  |  |  | 0.026 |  | 0.031 |  |  | 0.017 | 0.017 |
|  | b | 0.139 | 0.083 | 0.154 | 0.167 | 0.167 | 0.150 | 0.120 | 0.033 | 0.050 | 0.069 | 0.069 |  | 0.067 | 0.105 | 0.100 | 0.063 | 0.183 | 0.117 | 0.100 | 0.083 |
|  | c | 0.028 | 0.033 |  | 0.017 |  | 0.033 | 0.020 | 0.050 | 0.050 | 0.034 | 0.069 |  | 0.017 |  | 0.017 | 0.063 | 0.017 | 0.050 | 0.017 | 0.017 |
|  | d | 0.472 | 0.367 | 0.423 | 0.467 | 0.433 | 0.317 | 0.380 | 0.317 | 0.333 | 0.414 | 0.448 |  | 0.350 | 0.211 | 0.300 | 0.438 | 0.367 | 0.433 | 0.483 | 0.283 |
|  | e | 0.361 | 0.483 | 0.423 | 0.317 | 0.333 | 0.483 | 0.440 | 0.600 | 0.467 | 0.483 | 0.379 |  | 0.533 | 0.553 | 0.550 | 0.375 | 0.417 | 0.383 | 0.383 | 0.533 |
|  | f |  | 0.033 |  | 0.033 | 0.067 | 0.017 | 0.040 |  | 0.033 |  | 0.034 |  | 0.033 | 0.105 | 0.033 | 0.031 | 0.017 | 0.017 |  | 0.067 |
| Ps36 | a |  | 0.017 |  |  |  | 0.033 |  |  | 0.017 | 0.034 |  |  | 0.017 | 0.026 | 0.017 |  |  |  | 0.033 |  |
|  | b | 0.667 | 0.650 | 0.635 | 0.667 | 0.767 | 0.767 | 0.620 | 0.650 | 0.700 | 0.638 | 0.655 |  | 0.667 | 0.684 | 0.600 | 0.676 | 0.550 | 0.667 | 0.683 | 0.650 |
|  | c | 0.333 | 0.333 | 0.365 | 0.333 | 0.233 | 0.200 | 0.380 | 0.350 | 0.283 | 0.328 | 0.345 |  | 0.317 | 0.289 | 0.383 | 0.324 | 0.450 | 0.333 | 0.283 | 0.350 |
| Ps61 | a |  | 0.033 | 0.038 | 0.017 |  |  |  |  |  |  |  |  | 0.017 |  | 0.017 | 0.029 | 0.033 |  | 0.017 | 0.050 |
|  | b | 0.694 | 0.733 | 0.788 | 0.700 | 0.600 | 0.817 | 0.880 | 0.883 | 0.817 | 0.759 | 0.741 |  | 0.783 | 0.816 | 0.750 | 0.794 | 0.700 | 0.817 | 0.717 | 0.683 |
|  | c | 0.056 | 0.033 | 0.038 | 0.050 | 0.133 |  |  |  |  |  | 0.052 |  | 0.067 | 0.026 | 0.033 |  | 0.050 | 0.067 | 0.050 | 0.017 |
|  | d | 0.222 | 0.200 | 0.115 | 0.233 | 0.267 | 0.183 | 0.100 | 0.117 | 0.183 | 0.241 | 0.207 |  | 0.133 | 0.158 | 0.183 | 0.147 | 0.217 | 0.117 | 0.217 | 0.233 |
|  | e | 0.028 |  | 0.019 |  |  |  |  |  |  |  |  |  |  |  |  |  |  |  |  | 0.017 |
|  | f |  |  |  |  |  |  | **0.020** |  |  |  |  |  |  |  |  | 0.029 |  |  |  |  |
| Ps63 | a |  |  |  |  |  |  |  | 0.033 |  |  |  |  |  |  |  |  |  |  |  |  |
|  | b | 0.417 | 0.333 | 0.327 | 0.350 | 0.133 | 0.367 | 0.340 | 0.333 | 0.250 | 0.448 | 0.420 |  | 0.328 | 0.421 | 0.350 | 0.206 | 0.333 | 0.450 | 0.267 | 0.317 |
|  | c | 0.361 | 0.317 | 0.442 | 0.400 | 0.400 | 0.350 | 0.340 | 0.417 | 0.533 | 0.345 | 0.340 |  | 0.293 | 0.316 | 0.383 | 0.618 | 0.450 | 0.317 | 0.450 | 0.517 |
|  | d | 0.222 | 0.350 | 0.231 | 0.250 | 0.467 | 0.283 | 0.320 | 0.217 | 0.217 | 0.207 | 0.240 |  | 0.379 | 0.263 | 0.267 | 0.176 | 0.217 | 0.233 | 0.283 | 0.167 |
| Ps70 | a |  |  |  |  |  |  |  |  |  |  | **0.034** |  | 0.017 |  |  |  |  | 0.017 |  |  |
|  | b |  |  | 0.019 |  |  | 0.033 | 0.020 | 0.017 | 0.017 |  | 0.069 |  | 0.033 | 0.026 | 0.033 |  |  | 0.067 |  |  |
|  | c |  | 0.017 |  | 0.017 |  | 0.067 |  | 0.034 |  | 0.103 | 0.121 |  | 0.100 | 0.026 | 0.083 | 0.063 | 0.017 | 0.033 |  | 0.050 |
|  | d | 0.118 | 0.052 |  | 0.050 | 0.167 | 0.067 | 0.060 | 0.017 | 0.069 | 0.121 | 0.034 |  | 0.017 |  |  |  |  | 0.033 | 0.083 | 0.017 |
|  | e | 0.647 | 0.690 | 0.673 | 0.717 | 0.633 | 0.600 | 0.700 | 0.534 | 0.707 | 0.586 | 0.569 |  | 0.633 | 0.658 | 0.667 | 0.719 | 0.750 | 0.583 | 0.700 | 0.633 |
|  | f | 0.176 | 0.207 | 0.212 | 0.183 | 0.167 | 0.200 | 0.200 | 0.397 | 0.172 | 0.172 | 0.155 |  | 0.200 | 0.237 | 0.183 | 0.188 | 0.200 | 0.267 | 0.217 | 0.217 |
|  | g |  |  |  |  | **0.033** |  |  |  |  |  |  |  |  |  |  | 0.031 |  |  |  |  |
|  | h | 0.059 | 0.034 | 0.096 | 0.033 |  | 0.033 | 0.020 |  | 0.034 | 0.017 | 0.017 |  |  | 0.053 | 0.033 |  | 0.033 |  |  | 0.083 |
| Ps82 | a | 0.235 | 0.183 | 0.192 | 0.167 | 0.167 | 0.167 | 0.160 | 0.150 | 0.133 | 0.155 | 0.224 |  | 0.167 | 0.132 | 0.117 | 0.088 | 0.100 | 0.183 | 0.183 | 0.167 |
|  | b | 0.765 | 0.817 | 0.808 | 0.833 | 0.833 | 0.833 | 0.840 | 0.833 | 0.867 | 0.845 | 0.759 |  | 0.833 | 0.868 | 0.883 | 0.912 | 0.900 | 0.800 | 0.800 | 0.833 |
|  | c |  |  |  |  |  |  |  | 0.017 |  |  | 0.017 |  |  |  |  |  |  | 0.017 | 0.017 |  |
| Ps89 | a | 0.694 | 0.800 | 0.788 | 0.700 | 0.667 | 0.733 | 0.680 | 0.850 | 0.750 | 0.655 | 0.724 |  | 0.767 | 0.658 | 0.800 | 0.676 | 0.817 | 0.867 | 0.817 | 0.700 |
|  | b |  |  |  | 0.017 | 0.033 |  |  |  |  |  |  |  |  |  |  |  |  |  |  |  |
|  | c | 0.222 | 0.117 | 0.173 | 0.200 | 0.200 | 0.217 | 0.200 | 0.133 | 0.250 | 0.293 | 0.207 |  | 0.200 | 0.316 | 0.167 | 0.265 | 0.167 | 0.083 | 0.183 | 0.217 |
|  | d |  | 0.050 | 0.019 | 0.033 | 0.033 | 0.017 | 0.020 |  |  | 0.034 | 0.017 |  |  | 0.026 |  |  |  | 0.017 |  |  |
|  | e | 0.056 | 0.033 | 0.019 | 0.050 | 0.067 | 0.033 | 0.100 | 0.017 |  | 0.017 | 0.052 |  | 0.033 |  | 0.033 | 0.059 | 0.017 | 0.033 |  | 0.083 |
|  | f | **0.028** |  |  |  |  |  |  |  |  |  |  |  |  |  |  |  |  |  |  |  |
| Ps163 | a | 0.028 |  |  |  | 0.033 |  | 0.020 |  |  |  |  |  | 0.017 |  |  |  |  |  |  | 0.050 |
|  | b | 0.056 | 0.117 | 0.026 | 0.207 | 0.167 | 0.117 | 0.080 | 0.133 | 0.109 | 0.069 | 0.161 |  | 0.183 | 0.105 | 0.172 | 0.206 | 0.136 | 0.133 | 0.200 | 0.133 |
|  | c |  |  |  | **0.017** |  |  |  |  |  |  |  |  |  |  |  |  |  |  |  |  |
|  | d |  | 0.017 |  | 0.017 | 0.033 | 0.017 |  |  | 0.022 |  | 0.018 |  | 0.017 |  |  | 0.029 |  |  | 0.020 |  |
|  | e | 0.139 | 0.050 | 0.079 | 0.052 | 0.133 | 0.083 | 0.100 | 0.117 | 0.065 | 0.069 | 0.089 |  | 0.033 | 0.105 | 0.086 | 0.088 | 0.114 | 0.017 |  | 0.133 |
|  | f | 0.028 | 0.017 | 0.053 | 0.086 | 0.100 | 0.033 | 0.040 | 0.050 | 0.065 | 0.034 | 0.054 |  | 0.017 |  | 0.017 | 0.029 | 0.091 | 0.050 | 0.040 | 0.067 |
|  | g |  | 0.017 | 0.026 |  |  |  |  | 0.017 | 0.065 | 0.017 |  |  | 0.017 |  | 0.017 |  | 0.091 | 0.017 | 0.180 | 0.017 |
|  | h | 0.083 | 0.067 | 0.105 | 0.052 |  | 0.083 | 0.060 | 0.050 | 0.087 | 0.086 | 0.054 |  | 0.033 |  | 0.069 | 0.118 | 0.114 | 0.083 | 0.040 | 0.050 |
|  | i |  | 0.050 | 0.053 | 0.052 |  | 0.050 | 0.060 | 0.067 | 0.022 | 0.052 | 0.054 |  | 0.033 | 0.026 | 0.086 | 0.059 | 0.023 | 0.083 | 0.080 | 0.017 |
|  | j | 0.167 | 0.133 | 0.026 | 0.069 | 0.100 | 0.117 | 0.080 | 0.100 | 0.109 | 0.138 | 0.143 |  | 0.050 | 0.237 | 0.138 | 0.059 | 0.114 | 0.133 | 0.040 | 0.083 |
|  | k | 0.222 | 0.100 | 0.105 | 0.069 | 0.100 | 0.083 | 0.040 | 0.133 | 0.130 | 0.069 | 0.018 |  | 0.133 | 0.053 | 0.052 | 0.059 | 0.045 | 0.033 | 0.020 | 0.083 |
|  | l | 0.194 | 0.233 | 0.158 | 0.224 | 0.233 | 0.250 | 0.200 | 0.167 | 0.087 | 0.224 | 0.232 |  | 0.200 | 0.263 | 0.172 | 0.294 | 0.045 | 0.183 | 0.080 | 0.150 |
|  | m |  | 0.083 | 0.263 | 0.069 | 0.067 | 0.100 | 0.100 |  | 0.130 | 0.052 | 0.018 |  | 0.100 | 0.053 | 0.052 |  | 0.136 | 0.067 | 0.240 | 0.100 |
|  | n | 0.028 | 0.100 | 0.105 | 0.052 | 0.033 | 0.050 | 0.140 | 0.117 | 0.109 | 0.086 | 0.107 |  | 0.100 | 0.053 | 0.069 | 0.059 | 0.091 | 0.133 | 0.060 | 0.083 |
|  | o | 0.056 | 0.017 |  | 0.034 |  | 0.017 | 0.080 | 0.050 |  | 0.103 | 0.018 |  | 0.067 | 0.105 | 0.069 |  |  | 0.067 |  | 0.033 |
|  | p |  |  |  |  |  |  |  |  |  |  | **0.036** |  |  |  |  |  |  |  |  |  |
| Ps164 | a |  | **0.017** |  |  |  |  |  |  |  |  |  |  |  |  |  | 0.029 |  |  |  |  |
|  | b |  |  | 0.038 | 0.052 |  | 0.067 | 0.020 | 0.036 | 0.050 |  |  |  | 0.017 | 0.132 | 0.033 | 0.088 | 0.050 |  | 0.017 | 0.033 |
|  | c |  | 0.017 |  |  |  | 0.033 |  | 0.018 | 0.017 | 0.036 | 0.017 |  | 0.017 |  |  | 0.029 |  |  | 0.033 |  |
|  | d | 0.111 | 0.167 | 0.154 | 0.190 | 0.267 | 0.150 | 0.120 | 0.107 | 0.167 | 0.125 | 0.172 |  | 0.083 | 0.211 | 0.233 | 0.059 | 0.150 | 0.150 | 0.167 | 0.133 |
|  | e | 0.028 | 0.083 | 0.019 | 0.086 | 0.133 | 0.017 | 0.040 | 0.054 | 0.017 | 0.107 | 0.069 |  | 0.067 |  | 0.033 | 0.118 | 0.067 | 0.100 | 0.067 | 0.050 |
|  | f | 0.139 | 0.067 | 0.096 | 0.172 | 0.067 | 0.100 | 0.060 | 0.179 | 0.050 | 0.107 | 0.086 |  | 0.133 | 0.026 | 0.200 | 0.088 | 0.117 | 0.117 | 0.067 | 0.117 |
|  | g |  | 0.017 | 0.019 | 0.034 |  |  | 0.040 |  |  |  | 0.017 |  |  | 0.053 |  |  |  |  | 0.017 |  |
|  | h | 0.056 | 0.067 | 0.019 | 0.052 | 0.033 | 0.067 | 0.060 | 0.071 | 0.017 | 0.071 | 0.017 |  | 0.033 |  | 0.017 |  | 0.033 | 0.067 | 0.117 | 0.017 |
|  | i | 0.083 | 0.017 | 0.038 | 0.052 | 0.067 | 0.050 | 0.040 | 0.018 | 0.033 | 0.018 | 0.017 |  | 0.050 | 0.026 |  | 0.029 | 0.033 | 0.050 | 0.017 | 0.017 |
|  | j | 0.056 | 0.133 | 0.077 | 0.052 | 0.067 | 0.033 | 0.080 | 0.107 | 0.167 | 0.036 | 0.052 |  | 0.100 | 0.053 | 0.083 | 0.118 | 0.050 | 0.050 | 0.033 | 0.067 |
|  | k | 0.083 | 0.050 | 0.058 | 0.017 | 0.033 | 0.100 | 0.020 | 0.018 | 0.033 | 0.107 | 0.069 |  | 0.067 | 0.132 | 0.050 | 0.029 | 0.100 | 0.033 | 0.050 | 0.017 |
|  | l | 0.278 | 0.183 | 0.173 | 0.121 | 0.133 | 0.250 | 0.200 | 0.179 | 0.200 | 0.232 | 0.276 |  | 0.183 | 0.158 | 0.100 | 0.206 | 0.233 | 0.117 | 0.183 | 0.300 |
|  | m | 0.167 | 0.100 | 0.212 | 0.103 | 0.200 | 0.100 | 0.180 | 0.107 | 0.217 | 0.143 | 0.138 |  | 0.183 | 0.211 | 0.150 | 0.176 | 0.150 | 0.167 | 0.217 | 0.217 |
|  | n |  | 0.050 | 0.019 | 0.052 |  | 0.033 | 0.100 | 0.036 | 0.033 | 0.018 | 0.069 |  | 0.033 |  | 0.083 | 0.029 | 0.017 | 0.033 |  | 0.033 |
|  | o |  |  | **0.019** |  |  |  |  |  |  |  |  |  |  |  |  |  |  | 0.017 |  |  |
|  | p |  | 0.033 | 0.058 | 0.017 |  |  | 0.040 | 0.071 |  |  |  |  | 0.033 |  | 0.017 |  |  | 0.100 | 0.017 |  |
| lw_isotig07383 | a | 0.139 | 0.083 | 0.058 | 0.100 | 0.033 | 0.217 | 0.100 | 0.183 | 0.083 | 0.172 | 0.052 |  | 0.033 | 0.105 | 0.050 | 0.147 | 0.133 | 0.050 | 0.200 | 0.067 |
|  | b |  | 0.017 | 0.038 | 0.033 | 0.033 | 0.050 | 0.040 | 0.033 | 0.067 | 0.017 | 0.086 |  | 0.050 | 0.026 | 0.017 | 0.059 | 0.050 | 0.033 | 0.033 |  |
|  | c | 0.722 | 0.733 | 0.788 | 0.717 | 0.833 | 0.667 | 0.800 | 0.617 | 0.750 | 0.690 | 0.759 |  | 0.733 | 0.526 | 0.833 | 0.706 | 0.717 | 0.767 | 0.633 | 0.883 |
|  | d | 0.139 | 0.150 | 0.115 | 0.133 | 0.067 | 0.033 | 0.060 | 0.167 | 0.100 | 0.121 | 0.103 |  | 0.150 | 0.316 | 0.100 | 0.088 | 0.067 | 0.150 | 0.117 | 0.050 |
|  | e |  | 0.017 |  | 0.017 | 0.033 | 0.033 |  |  |  |  |  |  | 0.033 | 0.026 |  |  | 0.033 |  | 0.017 |  |

Note: the bold numbers are private alleles of native provenance populations; See Table 1 for population abbreviations.

.

**TABLE S3** The Bottleneck test of 11 native provenance populations of *Pinus sylvestris* var*. mongolica*.

| Population abbreviations | *P* values of tests and mode-shift for Bottleneck | | | |
| --- | --- | --- | --- | --- |
|  | Sign test | Standardized differences test | Wilcoxon signed-rank test | Mode shift |
| AES | 0.318 | 0.105 | 0.151 | NLS |
| CG | 0.381 | 0.075 | 0.519 | NLS |
| HHEJ | 0.543 | 0.426 | 0.970 | NLS |
| HM | 0.528 | 0.237 | 0.850 | NLS |
| JS | 0.378 | 0.349 | 0.850 | NLS |
| KLS | 0.550 | 0.420 | 0.910 | NLS |
| XK | 0.329 | 0.423 | 0.151 | NLS |
| MH | 0.551 | 0.222 | 1.000 | NLS |
| SBZ | 0.456 | 0.395 | 0.910 | NLS |
| TH | 0.476 | 0.486 | 0.791 | NLS |
| XLJ | 0.437 | 0.298 | 0.850 | NLS |

Notes: NLS, normal L-shaped distribution for Bottleneck; See Table 1 for population abbreviations.

**TABLE S4** Chi-square tests for Hardy-Weinberg equilibrium in 11 native provenance populations of *Pinus sylvestris* var*. mongolica*.

| Loci | AES | CG | HHEJ | HM | JS | KLS | MH | SBZ | TH | XK | XLJ |
| --- | --- | --- | --- | --- | --- | --- | --- | --- | --- | --- | --- |
| Ps8 | –0.015ns | 0.093^***^ | 0.236ns | 0.377^***^ | 0.376^*^ | 0.156ns | 0.228^***^ | 0.298^***^ | 0.302ns | 0.123ns | 0.181^*^ |
| Ps10 | –0.500^*^ | 0.042ns | –0.038ns | –0.304ns | 0.139ns | –0.023ns | –0.049ns | 0.101ns | –0.168ns | 0.504^**^ | 0.184ns |
| Ps23 | 0.025ns | –0.017ns | 0.254ns | –0.072ns | –0.096ns | 0.170ns | 0.071ns | 0.005ns | –0.161ns | –0.052ns | –0.231ns |
| Ps36 | –0.250ns | 0.070ns | 0.088ns | 0.250ns | –0.304ns | –0.168ns | 0.066ns | –0.245ns | 0.069ns | –0.067ns | –0.068ns |
| Ps61 | –0.316ns | –0.190ns | –0.063ns | –0.178ns | –0.210ns | –0.224ns | –0.115ns | –0.132ns | –0.224ns | –0.130ns | –0.278ns |
| Ps63 | 0.313ns | 0.099ns | 0.463^**^ | –0.018ns | –0.213ns | 0.095ns | 0.160ns | 0.351^***^ | 0.120ns | 0.405^*^ | 0.323ns |
| Ps70 | 0.338^**^ | 0.205ns | 0.219ns | 0.406^***^ | 0.016ns | 0.377^**^ | 0.227^*^ | 0.255^***^ | 0.332ns | 0.426^*^ | 0.234^***^ |
| Ps82 | 0.673^**^ | –0.224ns | 0.257ns | 0.040ns | –0.200ns | 0.040ns | 0.107ns | –0.179ns | 0.423^*^ | 0.079ns | 0.262ns |
| Ps89 | –0.196ns | 0.028ns | 0.004ns | 0.285ns | –0.048ns | –0.208ns | 0.015ns | –0.156ns | –0.156ns | –0.141ns | –0.123ns |
| Ps163 | 0.020ns | –0.065ns | 0.326^*^ | 0.052^*^ | –0.005ns | 0.007ns | 0.146ns | –0.090ns | 0.180ns | –0.092ns | 0.055ns |
| Ps164 | 0.077ns | 0.061^**^ | 0.077^*^ | –0.050ns | 0.125ns | 0.119ns | 0.003^*^ | 0.032ns | –0.061ns | –0.071ns | –0.055ns |
| lw_isotig07383 | –0.011ns | –0.157ns | 0.359^*^ | –0.166ns | –0.119ns | 0.140ns | –0.160ns | 0.163ns | –0.121ns | –0.078ns | –0.025ns |
| Mean | 0.013 | –0.005 | 0.182 | 0.052 | –0.045 | 0.040 | 0.058 | 0.033 | 0.045 | 0.076 | 0.038 |

Notes: ^***^, *P* < 0.001; ^**^, 0.001 ≤ *P* < 0.01; ^*^, 0.01 ≤ *P* < 0.05; ns, indicates not significant, level of significance of correlation coefficients; See Table 1 for population abbreviations.

**TABLE S5** Gene flow (*N*_m_, upper triangle) and genetic differentiation coefficient (*F*_ST_, lower triangle) between native provenance populations of *Pinus sylvestris* var. *Mongolica* based on SSR markers.

| Population | AES | CG | HHEJ | HM | JS | KLS | MH | SBZ | TH | XK | XLJ |
| --- | --- | --- | --- | --- | --- | --- | --- | --- | --- | --- | --- |
| AES |  | 26.055 | 27.143 | 38.142 | 18.448 | 27.069 | 19.955 | 12.498 | 20.798 | 34.925 | 22.696 |
| CG | 0.010 |  | 28.402 | 46.232 | 19.644 | 29.306 | 38.400 | 22.088 | 29.869 | 31.976 | 30.467 |
| HHEJ | 0.009 | 0.009 |  | 25.726 | 15.362 | 20.946 | 26.203 | 12.836 | 29.515 | 18.054 | 14.879 |
| HM | 0.007 | 0.005 | 0.010 |  | 25.407 | 29.159 | 30.890 | 15.483 | 30.521 | 39.257 | 34.930 |
| JS | 0.013 | 0.013 | 0.016 | 0.010 |  | 16.303 | 15.049 | 8.080 | 17.892 | 14.412 | 14.088 |
| KLS | 0.009 | 0.008 | 0.012 | 0.009 | 0.015 |  | 26.790 | 20.183 | 30.911 | 37.789 | 22.612 |
| MH | 0.012 | 0.006 | 0.009 | 0.008 | 0.016 | 0.009 |  | 19.755 | 33.910 | 27.851 | 25.883 |
| SBZ | 0.020 | 0.011 | 0.019 | 0.016 | 0.030 | 0.012 | 0.012 |  | 18.241 | 18.942 | 18.616 |
| TH | 0.012 | 0.008 | 0.008 | 0.008 | 0.014 | 0.008 | 0.007 | 0.014 |  | 26.595 | 19.546 |
| XK | 0.007 | 0.008 | 0.014 | 0.006 | 0.017 | 0.007 | 0.009 | 0.013 | 0.009 |  | 37.439 |
| XLJ | 0.011 | 0.008 | 0.017 | 0.007 | 0.017 | 0.011 | 0.010 | 0.013 | 0.013 | 0.007 |  |

Notes: See Table 1 for population abbreviations; there were no significant *F*_ST_ for each pair of populations after Bonferroni correction (Bonferroni corrected P-value, based on 1100 permutations, at ɑ=0.05 was 0.00091); the overall *F*_ST_ was 0.022 (95% CI 0.019–0.026); the overall *N*_m_ was 12.181 (95% CI 9.482–14.879).
